# A normative framework dissociates need and motivation in hypothalamic neurons

**DOI:** 10.1126/sciadv.ado1820

**Published:** 2024-11-06

**Authors:** Kyu Sik Kim, Young Hee Lee, Jong Won Yun, Yu-Been Kim, Ha Young Song, Joon Seok Park, Sang-Ho Jung, Jong-Woo Sohn, Ki Woo Kim, HyungGoo R. Kim, Hyung Jin Choi

**Affiliations:** ^1^ Department of Biomedical Sciences, Seoul National University College of Medicine, 103 Daehak-ro, Jongno-gu, Seoul 03080, Republic of Korea.; ^2^ Department of Anatomy and Cell Biology, Seoul National University College of Medicine, 103 Daehak-ro, Jongno-gu, Seoul 03080, Republic of Korea.; ^3^ Neuroscience Research Institute, Seoul National University College of Medicine, 103 Daehak-ro, Jongno-gu, Seoul 03080, Republic of Korea.; ^4^ Department of Intelligent Precision Healthcare Convergence, Sungkyunkwan University, Suwon 16419, Republic of Korea.; ^5^ Center of Neuroscience Imaging Research, Institute for Basic Science (IBS), Suwon 16419, Republic of Korea.; ^6^ Department of Brain and Cognitive Sciences, Seoul National University, Seoul, Republic of Korea.; ^7^ Department of Biological Sciences, Korea Advanced Institute of Science and Technology, Daejeon 34141, South Korea.; ^8^ Division of Physiology, Departments of Oral Biology and Applied Life Science, BK21 FOUR, Yonsei University College of Dentistry, Seoul, Korea.; ^9^ Department of Biomedical Engineering, Sungkyunkwan University, Suwon 16419, Republic of Korea.; ^10^ Wide River Institute of Immunology, Seoul National University, 101 Dabyeonbat-gil, Hwachon-myeon, Gangwon-do 25159, Republic of Korea.

## Abstract

Physiological needs evoke motivational drives that produce natural behaviors for survival. In previous studies, the temporally intertwined dynamics of need and motivation have made it challenging to differentiate these two components. On the basis of classic homeostatic theories, we established a normative framework to derive computational models for need-encoding and motivation-encoding neurons. By combining the model-based predictions and naturalistic experimental paradigms, we demonstrated that agouti-related peptide (AgRP) and lateral hypothalamic leptin receptor (LH^LepR)^ neuronal activities encode need and motivation, respectively. Our model further explains the difference in the dynamics of appetitive behaviors induced by optogenetic stimulation of AgRP or LH^LepR^ neurons. Our study provides a normative modeling framework that explains how hypothalamic neurons separately encode need and motivation in the mammalian brain.

## Teaser

Experimental and computational methods elucidate the neural substrates of need and motivation in hypothalamic neurons.

## INTRODUCTION

For survival, animals need to maintain energy homeostasis through diverse physiological processes and eating behaviors. Previous studies on eating behavior have attempted to identify psychological components in these ubiquitous and complex behaviors, and classic psychological theories have introduced the concepts of need and motivation (*1*–*5*). In the drive-reduction theory, the deviation of an internal state from the homeostatic set point creates motivational drive, which leads to eating behaviors (*6*, *7*). Although the theory provides conceptual understandings, it has been difficult to investigate the neural correlates of these homeostatic substrates.

Since these classic models often do not provide quantitative details, they may not be used to predict accurate neural dynamics during motivated behaviors. Modern theoretical computational models have proven to be highly useful in understanding brain areas, such as the cortex and the limbic system (*8*–*12*), but have not been used to explain homeostatic processes. Recently, studies have extended the reinforcement learning theory to include homeostatic perspectives (*13*). However, it is not known whether hypothalamic neurons, which are closely related to homeostatic eating behavior, can be normatively explained by theoretical models. Therefore, on the basis of these findings, we developed a computational model that can predict the temporal dynamics of neural activity and behaviors to distinguish the functional roles of different populations of hypothalamic neurons.

In the classic drive-reduction theory, the deviation of an animal’s internal state from the homeostatic set point is perceived as a “current deficit” (*7*). Historically, studies have demonstrated that animals not only monitor their current deficit but also predict future homeostatic state changes using external information (*14*–*19*). Since this external information helps to predict future changes, it acts as a “predicted gain (or loss),” which updates the “predicted deficit” in a feedforward manner. In other words, the brain computes a predicted deficit (*20*–*24*). In our study, we defined this predicted deficit as an animal’s overall “need” for food. Need acts as a homeostatic substrate in the brain, rapidly calculating requirements to return to the homeostatic set point in the future.

The predictive coding of deficits has many benefits, such as enabling preemptive preparation for efficient behavioral regulation and the avoidance of overcompensation (*25*). However, this alone might not be sufficient for adaptive behavior as animals also need a function that helps them choose and initiate appropriate behaviors. Previous models have successfully adapted an accumulation model to explain the dynamics of implicit motivation (*26*–*29*). For example, in the evidence accumulation model, behavior is initiated only when the accumulation of sensory information surpasses a certain threshold, allowing animals to make appropriate choices (*30*). Similarly, need accumulating into motivation could help animals produce efficient and persistent actions. We note that, depending on the presence of a specific target in a task, two distinct phases of motivations may drive behavior: (i) exploratory motivation, which drives searching behavior to find a goal, (ii) goal-directed motivation, which drives approach and consumption when animals are aware of a specified goal (*31*). In this study, we define “motivation” as goal-directed motivation, which is produced after the accessibility of a specific goal is acknowledged.

Using these models, we carefully examined whether and how the neural correlates of need and motivation for food are encoded in hypothalamic neurons. Agouti-related peptide (AgRP) neurons in the arcuate hypothalamus (ARC) are the major regulators of eating behaviors (*32*), although their functional role as a neural substrate for homeostasis has yet to be clarified. Previous studies have demonstrated that AgRP neurons activate to encode food need during fasting, responding to food-anticipatory signals (*33*–*35*). However, other studies argue that despite the deactivation of AgRP neurons, the residual firing of AgRP neurons directly encodes the food-related motivation responsible for initiating behaviors such as searching, foraging, consuming, or instrumental responses (*36*, *37*). In addition, we and other researchers have reported that leptin-receptor neurons in the lateral hypothalamus (LH^LepR^) are activated during food consumption (*38*, *39*). Similar to activating AgRP neurons, activation of LH^LepR^ neurons increases eating behaviors (*38*). Although both AgRP and LH^LepR^ neurons are known to be the major drivers of eating behavior, the distinct roles of these two neurons are not yet clarified.

In our study, we show that need defined in a predictive manner accurately explains both neural activities and behaviors. We show that the brain cannot just rely only on the need to generate efficient eating behaviors, and this issue can be resolved by an accumulation process. We applied our model to experimental data on the neural activity of AgRP and LH^LepR^ neurons [LH^LepR^ data and images from (*38*)], as well as behavioral activity traces during optogenetic activation of these neurons (For detailed framework, see Materials and Methods and Supplementary text). Our modeling results distinguish AgRP and LH^LepR^ neurons’ activities as the neural substrates for need and motivation, respectively.

## RESULTS

### Normative models theoretically dissociate neural substrates for eating behaviors

**Fig. 1.**
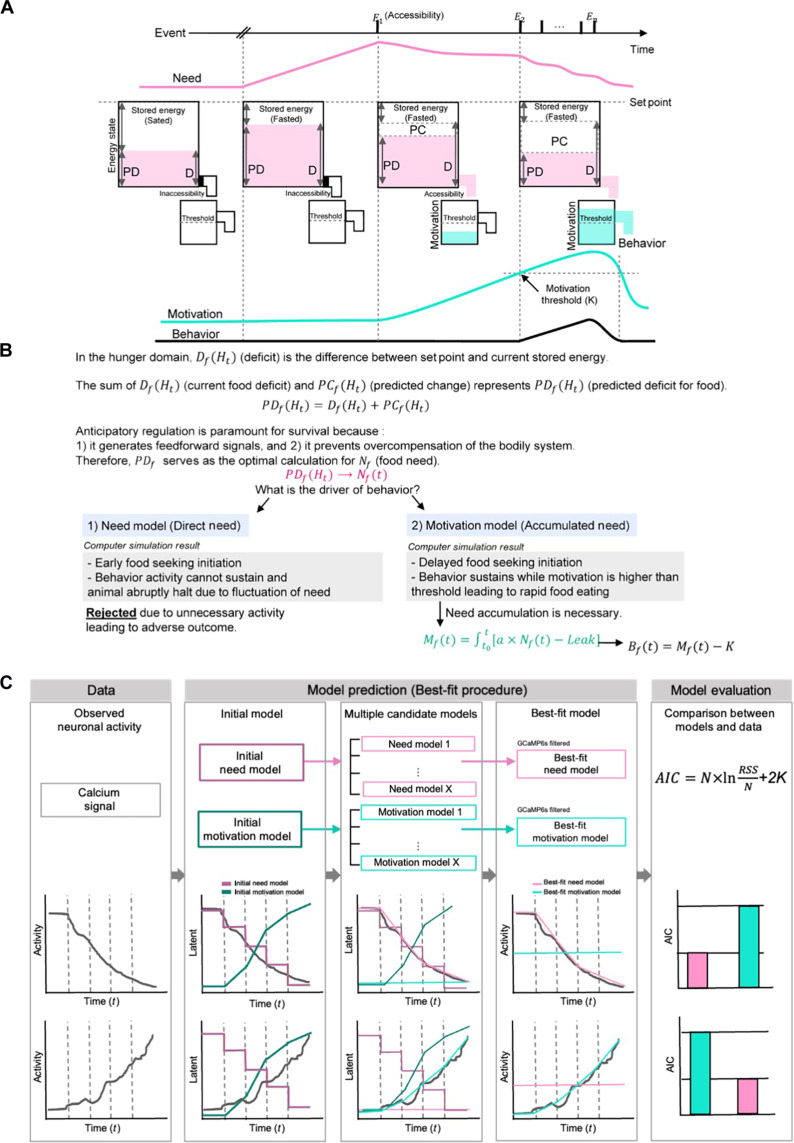
Outline of normative framework and computational analysis to dissociate need and motivation. (**A**) Schematic of the temporal dynamics of need and motivation. (**B**) Detailed description of the normative framework for (A). Simulation result shows that the motivation model (accumulated need) is the driver of behavior. (**C**) Pipeline of the model procedure in need and motivation models and schematic of the model-fit procedure. The latent values were obtained and convolved with the GCaMP6s kernel. To determine which model best explains the neuronal activity, Akaike information criterion (AIC) were obtained.

The food deficit (*D_f_*) is a function of homeostatic state (*H_t_*) at time *t*, which can be defined from the deviation of the homeostatic set point as below

$$D_{f}\left(H_{t}\right)=f\left(|h_{f}^{*}-h_{f,t}|\right)$$

where $h_{f}^{*}$ is the homeostatic set point and *h*_*f*,*t*_ is the homeostatic state at time *t*. For optimal survival, animals use external information to forecast future states. Therefore, a predicted change $\left[{\mathrm{PC}}_{f}\;(H_{t})\right]$ is defined as a change in future deficit predicted from external information. A predicted deficit $\left[{\mathrm{PD}}_{f}\;(H_{t})\right]$ is defined as the sum of current food deficit $D_{f}\;(H_{t})$ and all future predicted changes ${\mathrm{PC}}_{f}\;(H_{t})$ (Fig. 1A; see Supplementary text for detailed description) (*15*)

$${\mathrm{PD}}_{f}\left(H_{t}\right)=D_{f}\left(H_{t}\right)+{\mathrm{PC}}_{f}\left(H_{t}\right)$$

The predicted deficit should serve as the animal’s optimal choice to calculate the current food need $N_{f}\;(t)$ (afterward stated as need; Fig. 1, A and B)

$${\mathrm{PD}}_{f}\left(H_{t}\right)\to N_{f}\left(t\right)$$

Can need, $N_{f}\;(t)$, be the main driver for initiating eating behaviors? On the basis of our simulation results, $N_{f}\;(t)$ cannot be a driver of behavior since it delays food acquisition (fig. S1 and movie S1). Instead, motivation $\left[M_{f}\;(t)\right]$, which accumulates from need, serves as a more advantageous policy for generating rapid and efficient behavior (*30*). Inspired by previous studies of perceptual or foraging decision-making behavior models (*40*, *41*), we defined the intensity of food motivation as the accumulation of need with the product of integrated affecting factors (*a*) attenuated with a *Leak* (see Materials and Methods) (*30*, *42*, *43*)

$$M_{f}\left(t\right)=\int_{t_{0}}^{t}\left[a\times N_{f}\left(t\right)-\text{Leak}\right]$$

Since we model the target-dependent motivation, *t*_0_ is the moment when animals are granted access to the target. Behavior *B_f _*(*t*) is initiated when the motivation surpasses a threshold (*K*) (*26*–*29*)

$$B_{f}\left(t\right)=M_{f}\left(t\right)-K$$

Next, using a model-fitting analysis from our previous study, we established an analysis procedure to determine which hypothalamic neurons could be attributed to the need or motivation model, respectively (*10*). The need- and motivation-encoding models were initially defined on the basis of our framework above. Moment-by-moment time courses of need and motivation were generated using equations that were derived from each test (see Materials and Methods and Supplementary text for specific equations). The derived equations allowed us to fit the data with a minimum number of free parameters while incorporating the experimental events. We then convolved these latent variables with an ultrasensitive protein calcium sensor (GCaMP6s) kernel to account for the slow dynamics of the calcium sensor. By using this model-fit procedure (Fig. 1C, see Materials and Methods), we obtained a set of best-fit parameters that minimized the difference between the predicted and empirical neural responses.

### Experiments with predicted gain/loss dissociate the role of hypothalamic neurons

**Fig. 2.**
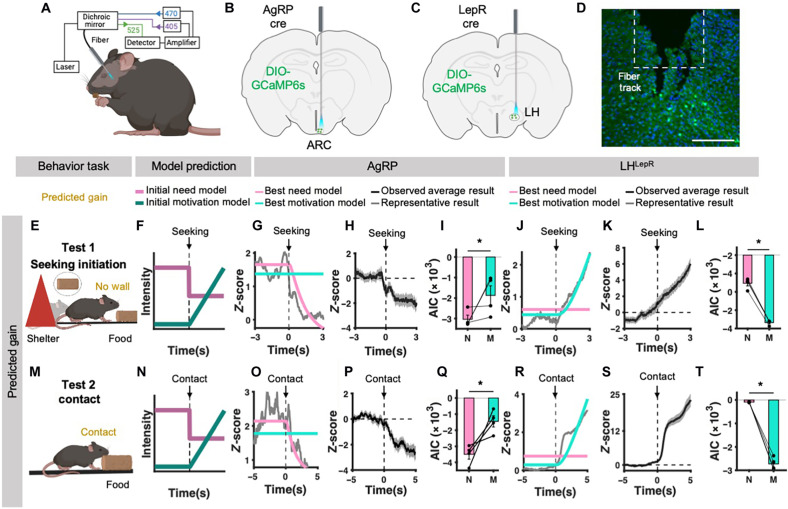
Events that induce predicted gain event dissociate AgRP and LH^LepR^ neurons as need and motivation, respectively. (**A**) Schematic of photometry experiment design. (**B** and **C**) Schematic of viral injection into ARC (B) and LH (C). (**D**) Image of GCaMP6s expression in LH^LepR^ neurons. Scale bar, 200 μm. (**E** and **M**) Schematic of tests. (E) Predicted gain test 1, (M) predicted gain test 2. (**F** and **N**) Schematic of initial model of need (pale violet-red) and motivation (teal) intensity in each test. Dotted line indicates the behavior of interest. (F) Seeking initiation moment. (N) Contact moment. (**G**, **J**, **O**, and **R**) Model fitting result between average neural activity from all trials in individual mice (gray) and best-fit need neural activity model (pink) or best-fit motivation neural activity model (turquoise) for each test. [(G) and (O)] Model fitting of AgRP neural activity. [(J) and (R)] Model fitting of LH^LepR^ neural activity. Vertical dotted line at 0 s indicates the moment of behavior of interest. (**H**, **K**, **P**, and **S**) Average trace of *z* score from all trials from individual mice. Vertical dotted line at 0 s indicates the moment of behavior of interest. [(H) and (P)] Neural activity from AgRP neurons in each test. [(K) and (S)] Neural activity from LH^LepR^ in each test. (**I** and **Q**) Quantification of AIC from AgRP neural activity and best-fit need neural activity model (pink) or best-fit motivation neural activity model (turquoise). (**L** and **T**) Quantification of AIC from LH^LepR^ neural activity and best-fit need neural activity model (pink) or best-fit motivation neural activity model (turquoise). [(E) to (L)] Data from predicted gain test 1 (AgRP, LH^LepR^ *N* = 4, 4; trials = 46, 67). [(M) to (T)] Data from predicted gain test 2 (AgRP, LH^LepR^ *N* = 4, 4; trials = 32, 32). *P ≤ 0.05, **P ≤ 0.01, and ***P ≤ 0.001.

On the basis of our models, we investigated whether the neural activity of hypothalamic neurons best represents the properties of need or motivation. To measure neural activity, AgRP-cre and LepR-cre mice were injected with cre-dependent adeno-associated virus (AAV) carrying GCaMP6s, and an optic fiber was implanted in the ARC or the LH, respectively (Fig. 2, A to D).

We first conducted two experiments in fasted mice to identify whether and which of the hypothalamic neurons encode need or motivation. These experiments were specifically designed to evoke events that induce the predicted gain of a food target: (ii) seeking initiation (predicts food discovery) and (ii) contact (predicts nutrient absorption). According to our models, when animals begin seeking and consuming food, predicted gain events such as seeking initiation and contact bring about changes in their future deficit. During this process, the possibility of achieving the goal of consuming food becomes clearer, alleviating the future deficit. This is equivalent to a decrease in the sum of discounted deficit, *SDD*_π_(*H_t_*) (eq. S3), leading to a reduction in the predicted change (eq. S4). Consequently, the predicted deficit decreases (eq. S5), indicating a reduction in need at that moment (eq. S13). Simultaneously, there is an increase in motivation due to the accumulation of need (Fig. 2, E, F, M, and N). In the seeking initiation experiment (Fig. 2, E and F), our neural recordings showed that the AgRP neural activity started to decrease at the voluntary seeking initiation event, while the neural activity of LH^LepR^ started to increase (Fig. 2, G, H, J, and K). Comparisons of the goodness of fit revealed that the AgRP and LH^LepR^ neural activity was significantly more consistent with the best-fit need model and motivation models, respectively (Fig. 2G, I, J, and L). Likewise, in the contact experiment (Fig. 2, M and N), our neural recordings showed that AgRP neural activity started to decrease at the food contact, while the neural activity of LH^LepR^ started to increase (Fig. 2, O, P, R, and S), which is consistent with the best-fit need model and motivation model, respectively (Fig. 2, O, Q, R, and T).

**Fig. 3.**
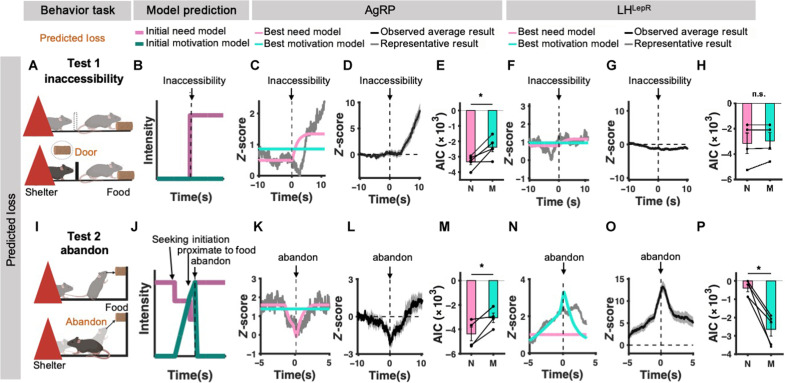
Events that induce predicted loss dissociate AgRP and LH^LepR^ neurons as need and motivation, respectively. (**A** and **I**) Schematic of tests. (A) Predicted loss test 1, (I) predicted loss test 2. (**B** and **J**) Schematic of initial model of need (pale violet-red) and motivation (teal) intensity. Dotted line indicates the behavior of interest. (B) Inaccessibility moment, (J) abandon moment. (**C**, **F**, **K**, and **N**) Model fitting result between average neural activity data from all trials in individual mice (gray) and best-fit need neural activity model (pink) or best-fit motivation neural activity model (turquoise). Vertical dotted line at 0 s indicates the moment of behavior of interest. [(C) and (K)] Model fitting of AgRP neural activity in each test. [(F) and (N)] Model fitting of LH^LepR^ neural activity. (**D**, **G**, **L**, and **O**) Average trace of *z* score from all trials from individual mice. Vertical dotted line at 0 s indicates the moment of behavior of interest. [(D) and (L)] Neural activity from AgRP neurons in each test. [(G) and (O)] Neural activity from LH^LepR^ in each test. (**E** and **M**) Quantification of AIC from AgRP neural activity and best-fit need neural activity model (pink) or best-fit motivation neural activity model (turquoise) in each test. (**H** and **P**) Quantification of AIC from LH^LepR^ neural activity and best-fit need neural activity model (pink) or best-fit motivation neural activity model (turquoise) in each test. [(A) to (H)] Data from predicted loss test 1 (AgRP, LH^LepR^ *N* = 5, 4; trials = 75, 60). [(I) to (P)] Data from predicted loss test 2 (AgRP, LH^LepR^ *N* = 4, 5; trials = 20, 17). n.s., not significant, *P ≤ 0.05, **P ≤ 0.01, and ***P ≤ 0.001.

To further distinguish the identity of hypothalamic neurons, we investigated neural activity during events that induce the predicted loss of a food target: (i) inaccessibility (predicts prolonged starvation) and (ii) abandon (predicts food loss). In the inaccessibility experiment, according to our models, the predicted loss produced an increase in need as predictions shifted from the expectation of achieving a goal to the expectation of its unattainability, resulting in a rise in future deficit (eq. S3). However, the motivation will not change because the inaccessibility blocks access to the goal (Fig. 3, A and B, and eq. S14). The neural activity of AgRP increased at the inaccessibility event, whereas the neural activity of LH^LepR^ remained stable (Fig. 3, C, D, F and G). These results were consistent with the best-fit need model and motivation model, respectively (Fig. 3, C, E, F, and H). In the abandon experiment, according to our models, predicted loss produces an increase in need and induces zero motivation (Fig. 3, I and J, and eq. S14). Consistent with the need model, the neural activity of AgRP increased at the moment of eating abandon and showed sustained activity (Fig. 3, K to M). The neural activity of LH^LepR^ decreased, consistent with the motivation model (Fig. 3, N to P). Similarly, model-fit results of individual trials from each mouse confirmed the same conclusion in a similar behavior paradigm upon voluntarily abandoning eating after seeking behavior (fig. S2). Furthermore, temporal dynamics of AgRP and LH^LepR^ neural activity were shown to be distinctive by unbiased dimensionality reduction methods (figs. S3 and S4 and movie S2).

**Fig. 4.**
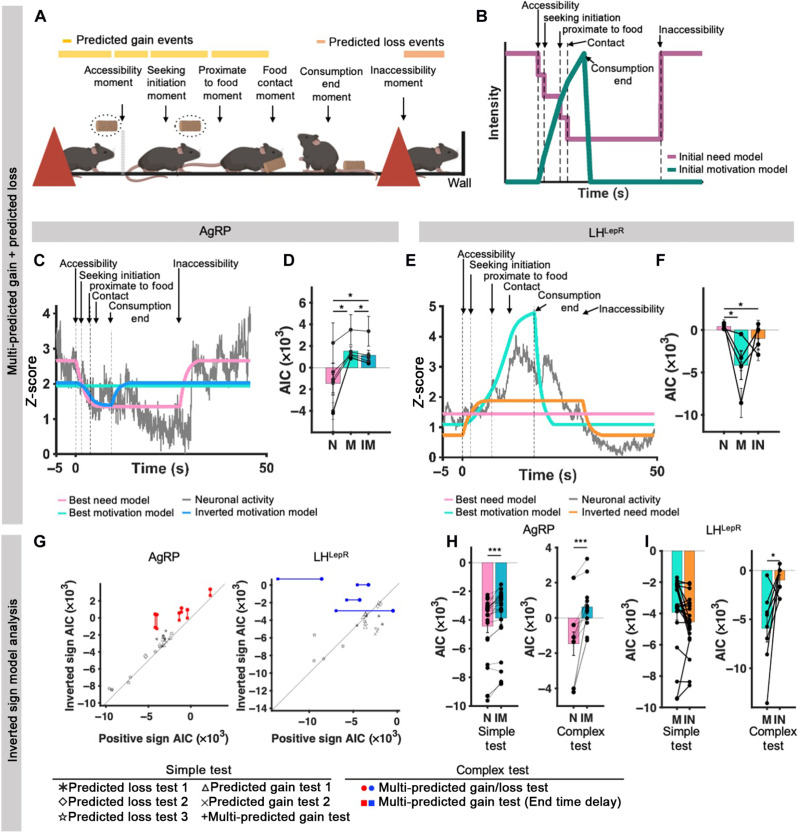
Events that induce multi-predicted gain/loss and theory-driven models dissociate AgRP and LH^LepR^ neurons as need and motivation, respectively. (**A**) Schematic of behavioral paradigm of events that induce multi-predicted gain/loss test. (**B**) Schematic of initial model of need (pale violet-red) and motivation (teal) intensity. (**C** and **E**) Representative cross-validated model fitting result between single trial neural activity data (normalized *z* score, gray) and best-fit need neural activity model (pink), best-fit motivation neural activity model (turquoise), best-fit inverted motivation model (blue), or best-fit inverted need model (orange) from an individual trial. Error bar in each point represents SEM for test dataset in each individual mice. (C) Neural activity from AgRP neurons (*N* = 6, trials = 56). (E) Neural activity from LH^LepR^ neurons (*N* = 4, trials = 50). (**D** and **F**) Quantification of AIC between neural activity and best-fit need neural activity model (pink), best-fit motivation neural activity model (turquoise), best-fit inverted motivation model (blue), or best-fit inverted need model (orange). (D) AIC quantification from AgRP neurons (*N* = 6), (F) AIC quantification from LH^LepR^ neurons (*N* = 4). (**G**) Scatter plot of inverted sign AIC and positive sign AIC. (**H** and **I**) Quantification of AIC in simple tests and complex tests in AgRP and LH^LepR^ neurons. Simple test indicates simple event tests, and complex test indicates multi-event tests. Data are means ± SEM. See table S2 for statistics. Source data are provided as a source data file. The schematics in (A) was created using BioRender. *P ≤ 0.05, **P ≤ 0.01, and ***P ≤ 0.001.

Although the above results look quite clear, one may argue that other “control models” should be tested. LH^LepR^ neurons might be potential anatomical downstream targets of AgRP neurons as they are affected by neuropeptide Y (*36*) and AgRP neurons are mostly GABAergic (*42*). If AgRP neurons send strong GABAergic projections to LH^LepR^ neurons, LH^LepR^ activity could encode need but with an inverted sign. Similarly, AgRP neurons might encode motivation with an inverted sign. In some relatively simple experiments, these “inverted sign” models explained data reasonably well. While the need model can still explain AgRP activities better than the inverted motivation model, the motivation model is on par with the inverted need model (Fig. 4).

To address this issue, we focused on the fact that natural eating behaviors involve multiple sequential events that induce dynamic and consecutive predicted changes in energy homeostasis. We conducted a multi-predicted gain/loss test, which contains events that induce both predicted gain and loss of a targeted food (Fig. 4A). In the task, fasted mice first conducted events that induced multi-predicted gain. Mice then showed consumption end (an event that induces the completion of predicted gain) and were subjected to an event that induced predicted loss (inaccessibility). During the test, the mice were well conditioned, traversing through the chamber without hesitation to the food with no abrupt stops.

According to our models, need would decrease whenever a predicted gain occurred and would show a rebound to increase when inaccessibility occurred. Conversely, motivation would start to increase at the accessibility moment and then decline sharply after consumption, remaining unchanged at the inaccessibility event (Fig. 4B). Because of the self-paced nature of the task, the timing of these sequential events varied greatly across trials. Therefore, we performed model fitting using individual trials (fig. S5 and table S1; model fits for Figs. 2 and 3 used averaged activity across trials). AgRP and LH^LepR^ neural activities were consistent with the best-fit need and motivation model, respectively (Fig. 4, C to F; fig. S5; and movie S3). In addition, measuring the precise moment for the event of consumption end is challenging because of the residual food in the animal’s mouth. To compensate for this, we performed an additional model fit by incorporating a delay of 0 to 8 s from the measured consumption end. The model with the added delay showed higher performance compared to the model without it (figs. S5 and S6). We further compared the need and motivation model with the inverted motivation and need model, respectively (Fig. 4, D, F, and G). The complex dynamics of data in the multiple gain/loss event tests allowed us to distinguish need and motivation from the inverted counterparts (Fig. 4, H and I). We further did cross-validation studies to verify that our model could generalize without overfitting. We could still observe that AgRP and LH^LepR^ neural activities were consistent with the best-fit need and motivation model, respectively (Fig. 4, C to F, and figs. S5 and S6). These results show that data from more naturalistic experiments have the power to distinguish models with seemingly similar predictions.

We further examined whether our model could predict neural activity while the animals underwent transitions of states through multiple events instead of a single event, as previously reported (*37*). As expected, the best-fit for AgRP neural activity was the need model, which decreased at each predicted gain rather than single-event decrease models (fig. S7). Similarly, for LH^LepR^ neurons, the best-fit was the multi-event motivation model (fig. S8). These results indicate that when animals learn a sequence of food-predicting events, the neurons predicting food intake show a cascade of shifts, rather than one big shift, as the animals perform the task. Collectively, these results demonstrate that the neural activity of AgRP and LH^LepR^ is temporally dissociable and consistent with need and motivation, respectively.

### Theory-driven models explain behaviors evoked by activation of hypothalamic neurons

**Fig. 5.**
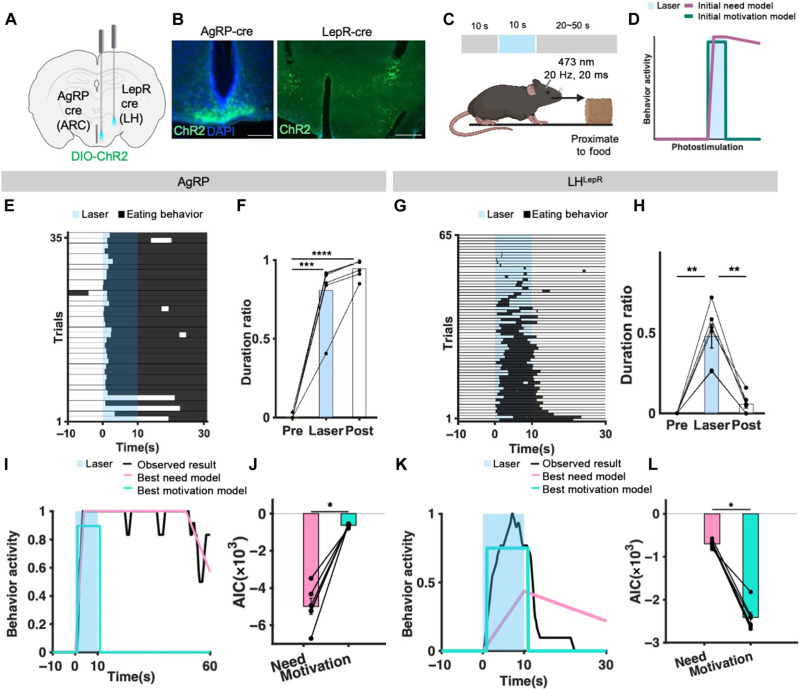
Theory-driven models dissociate behavior activity evoked from AgRP and LH^LepR^ neural activation as need-evoked behavior and motivation-evoked behavior, respectively. (**A**) Schematic of virus injection into the target site. (**B**) Representative image validates ChR2 expression in AgRP (scale bar, 200 μm) and LH^LepR^ neurons (scale bar, 500 μm). (**C**) Schematic of behavioral paradigm and event. (**D**) Schematic of initial model of behavior activity evoked from need (pale violet-red) and motivation (teal) neuronal activation. Duration of optogenetic stimulation colored in blue. (**E**) Raster plot of eating behavior (black) shown for each individual trial from all AgRP mice. (*N* = 6, trials = 36). (**F**) Behavior quantification of behavior duration ratio from (E). (**G**) Raster plot of eating behavior (black) shown for each individual trial from LH^LepR^ mice (*N* = 4, trials = 65). (**H**) Behavior quantification of behavior duration ratio from (G). (**I** and **K**) Model fitting of behavior activity between behavior activity data from AgRP or LH^LepR^ neuronal activation (black) and behavior activity prediction from best-fit need neuron activation (pink) or best-fit motivation neuron activation (turquoise). (I) Behavior activity from AgRP neuronal activation (one mouse, six trials). (K) Behavior activity from LH^LepR^ neuronal activation (1 mouse, 10 trials). (**J** and **L**) Quantification of AIC between behavior activity from AgRP or LH^LepR^ neuronal activation and best-fit need behavior activity model (pink) or best-fit motivation behavior activity model (turquoise). (J) Quantification of AIC from AgRP neuronal behavior activation (*N* = 6). (L) Quantification of AIC from LH^LepR^ neuronal behavior activation (*N* = 6). Data are means ± SEM. See table S2 for statistics. Source data are provided as a source data file. The schematics in (A) and (C) were created using BioRender. *P ≤ 0.05, **P ≤ 0.01, and ***P ≤ 0.001.

Next, we investigated whether the manipulation of these neurons affects behaviors as predicted by our models. We injected channelrhodopsin (ChR2) virus, implanted optic fibers in the ARC or LH (Fig. 5, A and B), and tested ad libitum mice in an eating-evoking test, activating target neurons for 10 s (Fig. 5C).

Our models predict that optogenetic activation of need and motivation neurons would result in different temporal dynamics of behavior (Fig. 5D). If need neurons were activated, the need would rapidly surge to its highest intensity and then gradually accumulate to generate motivation. Once the accumulated motivation surpasses the behavior initiation threshold, it would produce eating behaviors. When activation of the need neuron terminates, the intensity of the need would return to null; however, the motivation would gradually decrease. The behavior would continue until the motivation decreases below the behavior threshold. On the other hand, if the motivation neurons were activated, the motivation level would surge and immediately surpass the behavior threshold to produce eating behavior. When motivation neuron activation terminates, the sudden drop in motivation would lead to the immediate cessation of the eating behaviors (Fig. 5D).

The behavior induced by AgRP neural activation significantly sustained even after the termination of the neuronal activation (Fig. 5, E and F). On the contrary, the behavior induced by LH^LepR^ neural activation terminated immediately upon neuronal deactivation (Fig. 5, G and H), consistent with previous findings (*38*, *44*). When we fitted the temporal dynamics of behavioral responses evoked by activating hypothalamic neurons with our models, behavior activity induced by AgRP and LH^LepR^ neuronal activation was indeed significantly closer to the best-fit need model and best-fit motivation model, respectively (Fig. 5, I to L, and movie S4). Collectively, our results disambiguate the roles of hypothalamic neurons, dissociating AgRP neurons as need neurons and LH^LepR^ neurons as motivation neurons (fig. S9 and movie S5).

## DISCUSSION

In this study, we demonstrate that the role of the hypothalamic AgRP and LH^LepR^ neurons can be explained by the models of need and motivation in eating behaviors. By incorporating events, we were able to differentiate between need and motivation in recorded neural activities. Through model-fit analysis, we revealed that the AgRP and LH^LepR^ neurons encode need and motivation, respectively. Moreover, by manipulating the two types of neurons, we observed distinct temporal patterns in eating behaviors and our behavior activity models accurately predicted these differences. Collectively, our findings shed light on the neural substrates of distinct homeostatic constructs in eating behavior.

### Normative framework to investigate the neural substrate of eating behaviors

Analysis based on a normative modeling framework has proven to be a valuable approach in some computational fields as it reveals additional information of the relationship between subjects (*45*). Recently, normative mathematical models connecting homeostasis and brain reward systems have been suggested (*13*). However, exactly how it can be applied to temporal dynamics of neurons has not been shown. In our study, we have demonstrated that the functions of AgRP and LH^LepR^ neurons can be normatively explained, using models derived from recent theories of homeostasis (*13*). Our study used the normative framework to precisely elucidate the neural substrates of need and motivation within the heterogeneous hypothalamic neuronal populations, namely, AgRP and LH^LepR^ neurons. This normative framework provides a temporal dynamic understanding of how these neurons function at a comprehensible level.

### Computational approaches for understanding the neural substrates of homeostatic components

The original drive-reduction theory lacked quantitative details, making it challenging to identify neural substrates of homeostatic latent variables in the brain (*6*). Moreover, the smooth internal transition of need and motivation in natural conditions further complicated their identification. In our study, we examined low-dimensional components and embeddings to confirm that representative features differ between the two populations. We then investigated the nature of these populations using theory-driven models by directly modeling latent variables and adjusting the slow dynamics of measured signals (Fig. 1C, GCaMP6s filter). Our model successfully accounted for both trial-averaged activities and trial-to-trial activities (see table S1).

One may argue, however, that since these analyses only show correlations between latent variables and neural activities, the study has critical limitations and lacks the evidence of causality. To address this issue, we conducted neuronal manipulations. Our optogenetic manipulation influenced behaviors with the temporal characteristics predicted by our models. The high temporal resolution of optogenetic manipulation allowed us to confirm the causality of our model, the accumulation of need. Our integrative approach could help decipher the role of other hypothalamic neurons, encoding different homeostatic need and motivation features (thermoregulation, thirst, osmolarity, and competing need/motivation situations) to uncover hidden latent features and explain behaviors. For instance, neurons that respond to water in the subfornical organs (*16*) and the relevant circuits can be investigated to reveal their normative roles. Our model could also potentially provide insight into competing need situations. A recent study showed that animals exhibit stochastic choice between food and water and persist in the chosen behavior (*46*). The persistent activity could be explained by the accumulation of need into motivation. Once motivation has accumulated to surpass a behavior threshold, it takes time for motivation to decay and fall below the behavior threshold. This results in persistent behavior instead of a rapid transition of behavior. However, as our models have only been confirmed in the realm of feeding, other physiological conditions in different homeostatic contexts will need careful consideration of their different scenarios when extrapolating our models. It is also notable that optogenetic stimulation does not imitate physiological conditions and could push neurons outside their physiological range. This limitation requires caution when extrapolating our optogenetic results to natural environments.

Our model also provides insight into well-established concepts such as exploration and exploitation, goal-directed and habitual processes, and model-free and model-based processes. One way to distinguish between exploration and exploitation is whether the subject knows the location of a resource (*47*). In our study, mice were well conditioned to know the location of food in the environment. Our model was designed on the basis of the assumption that the mice would exploit the given information to obtain food. Further studies would be required to establish a framework for models that can explain these exploratory motivational processes.

In goal-directed behaviors, changing the value of the final goal (e.g., devaluation) directly affects the behavior, whereas the animals keep pressing levers for food regardless of devaluation once habits are formed (*48*, *49*). Goal-directed behaviors are frequently explained by a model-based control system that evaluates the outcome online based on the model of the environment, and habitual behaviors are executed by a model-free system (*50*). In principle, our model was built on the basis that mice would use a model-based strategy where mice have cognitively established how to obtain food in a given environment (*51*). AgRP neurons do not respond to food in fed mice, and LH^LepR^ neural activity changes according to fasted or fed states during food zone entry, which suggests that their neural activity might be a goal-directed response (*52*, *53*). However, it is possible that mice could use a model-free strategy throughout the association of certain sensory cues and predicted change. As shown in humans, mice may use the combination of two systems in our tasks. Further studies would be needed to elucidate whether and how the two systems are involved in the activities of AgRP and LH^LepR^ neurons.

Our computer simulations demonstrated that the accumulation of need is necessary for the animal’s survival. The level of accumulation would depend on the foraging environment. For example, in an environment where both food and competitors are abundant, little accumulation ensures early movement initiation and increases the probability of obtaining food. Organisms might have evolved to use the optimal level of accumulation for the given environment. This evolutionarily driven shift toward motivation-guided behavior highlights the intricate interplay between physiological need and the motivation process, ultimately shaping survival strategies.

### Deciphering temporal neural activity in hypothalamic neurons

During events that induce predicted loss (inaccessibility and abandon moments), AgRP neural activity showed a rapid increase within seconds. This expands our knowledge of AgRP neural activity, rapidly increasing upon cognitive events in adult mice, consistent with the results observed in neonatal mice being isolated from the nest and mice responding to hidden peanut butter smells, all events that induce predicted loss (*52*, *54*). Along with their nature of responding to voluntary food-seeking initiation, the predictive nature of AgRP neurons suggests that more cognitive components may be involved in the hypothalamic and subcortical processes related to hunger.

Our optogenetic results demonstrate that the temporal dynamics of behavior activity induced by AgRP neural activation are consistent with need-induced behavior activity models. Previous optogenetic studies on AgRP neurons perplexed the temporal dynamics of neural activity and behavior outcomes due to averaging methods (*37*, *44*, *55*). Our study, along with previous studies, showed that mice exhibit sustained eating behaviors after terminating the AgRP neural activation (*44*). Furthermore, in a previous study, optogenetically activating AgRP neurons in mice that had to search for a small food in a cage led to a substantial latency in initiating eating behavior (*55*). Conversely, in our study, AgRP neurons were activated when the animals were proximate to food (movie S4). Although our study could not observe a similar substantial latency to eat, because of the short timing to initiate eating behavior, we speculate that this phenomenon could be related to our model. The difference in experimental settings might have had an effect on the integrating affecting factor or behavior threshold, making a difference in the dynamics of the accumulation of motivation (Fig. 1 and Supplementary Text). Further studies will be needed to directly address the latency of initiating eating behavior due to the accumulation of need into motivation. Collectively, our model of need accumulation to generate motivation can explain the mechanism of the sustained behaviors after termination of AgRP neural activation.

Previous studies have not fully accounted for the multitude of dynamic events that can influence the activity of LH^LepR^ neurons (*39*, *56*). Past studies demonstrated that LH^LepR^ neurons reinforce lever pressing, have rewarding properties (*56*), and initiate seeking for food (*38*). Some results on consummatory behaviors for food have shown controversial results. While one study showed no change in consummatory behavior (*56*), our prior study showed that consummatory behavior could be evoked once seeking behavior was complete (*38*). In our experimental paradigm, LH^LepR^ neural activity increased at the accessibility moment and decreased at the inaccessibility moment. Our optogenetics results comprehensively demonstrate that the immediate incline and decline of behavior activity are time-locked to stimulation, consistent with our models. Further investigation is needed to account for fitting seeking and consummatory motivations, as LH^LepR^ neurons have been reported to have seeking and consummatory neuronal subpopulations (*38*). Collectively, these results demonstrate that LH^LepR^ neurons encode food-directed motivation.

Our framework also considers how metabolic change can increase need by changing the current deficit (Fig. 1A). An animal continuously uses energy to sustain life, which leads to an increase in the current deficit. These current deficit signals possibly come from vagal afferent signals through the nucleus of the solitary tract, which are known to project to the hypothalamus, delivering metabolic signals of hunger or satiety (*57*). As animals show higher motivation for food during hunger states, our framework could aid in establishing experimental paradigms for future studies to investigate the effect of metabolic change in regulating need and motivation. Overall, our work unravels how the two distinct neuronal populations in the brain rapidly orchestrate the two neural substrates of homeostatic behaviors, need and motivation, to optimize survival of the animals.

## MATERIALS AND METHODS

### Animals

All experimental protocols were approved by the Seoul National University Institutional Animal Care and Use Committee and were performed as per the health guidelines for the care and use of laboratory animals from the Seoul National University (SNU-200218-4-6). The mice were housed on a 08:00 to 20:00 light cycle, with standard mouse chow and water provided ad libitum, unless otherwise noted. Behavioral tests were performed in the behavior chamber during the light cycle. Adult male mice (at least 8 weeks old) were used for all behavioral experiments from the following Cre recombinase–expressing mouse lines: LepR-Cre, JAX stock no.008320, a gift from K.W.K., Yonsei University; AgRP-IRES-Cre, JAX stock no. 012899, a gift from J.-W.S., Korea Advanced Institute of Science and Technology, were used.

### Stereotaxic virus injection

Cre recombinase–expressing mouse lines and Cre-dependent AAV vectors were used. AgRP-ires-Cre mice were injected with the virus to study ARC, and LepR-Cre mice were injected with the virus to study LH. The mice were anesthetized with xylazine (20 mg/kg) and ketamine (120 mg/kg) in saline and placed in a stereotaxic apparatus (KOPF or Stoelting).

For calcium imaging experiments (fiber photometry), a pulled glass pipette was inserted into the target site of the ARC (300 nl total) at the coordinates anterior-posterior (AP), −1.3 mm; medial-lateral (ML), ±0.2 mm; dorsal-ventral (DV), 5.85 mm, from the bregma for AgRP-cre mice, and the LH (400 nl total), at the coordinates AP, −1.5 mm; ML, ±0.9 mm; DV, 5.25 mm, from the bregma for LepR-cre mice. After 10 min, the GCaMP6 virus (AAV1.Syn.Flex.GCaMP6s.WPRE.SV40, Addgene; titer 1.45 × 10^13^ genome copies/ml) was injected unilaterally for 10 min using a micromanipulator (Nanoliter 2010). The glass pipette was kept at the target site for 10 min following infusion; it was then withdrawn gradually. For optogenetic experiments, opsin virus (AAV5.EF1a.DIO.hChR2 (H134R). EYFP, Addgene; titer 2.4 × 10^13^ genome copies/ml) was unilaterally injected into the ARC (300 nl; AP, −1.3 mm; ML, ±0.2 mm; DV, 5.85 mm, from the bregma) for AgRP-ires-mice and bilaterally injected into the LH (400 nl; AP, −1.5 mm; ML, ±0.9 mm; DV, 5.25 mm, from the bregma) for LepR-cre mice.

### Optical fiber insertion

An optical fiber was inserted on the same day of injecting the virus. The optical fiber was implanted 30 min after injecting the virus to prevent backflow. For fiber photometry experiments, a ferrule-capped optical cannula [400-μm core, numerical aperture (NA) 0.57, Doric Lenses, MF2.5, 400/430–0.57] was placed unilaterally 0 to 50 μm above the site of virus injection as previously described; it was attached to the skull with Metabond cement (C&B Super Bond). For optogenetic activation of AgRP neurons, a unilateral optical fiber (200-μm core, NA 0.37, Doric Lenses, ZF1.25_FLT) was implanted 100 μm above the ARC injection site and secured to the skull with Metabond cement. For optogenetic manipulation of the LH^LepR^ neurons, optic fiber was implanted bilaterally 100 to 500 μm above the site of LH injection at a 10° angle from the vertical in the lateral to the medial direction; it was affixed to the skull with Metabond cement. Before and after the surgery, dexamethasone, ketoprofen, and cefazolin were administered for postoperative care. All mice were recovered in their cages for at least 2 weeks before conducting the behavioral experiments. The mice were handled for 5 days to relieve stress and acclimated to the behavior chamber for 30 min before testing.

### Behavior tests

#### Animal conditioning

Before the experiments, the mice were habituated to the experimental chambers, and fiber handling was conducted for 3 days. The mice were fasted 80 to 90% of the body weight in the ad libitum state. Trials were omitted manually if time between events were too long than average as a large variance between events made average traces difficult to comprehend during analysis.

#### Predicted gain test 1

A shelter was placed, and an electrical shock was given as punishment in a square chamber, as previously reported (*38*). All reward-associated cues (e.g., visual or sound cues) were eliminated to measure voluntary seeking behavior initiation. During the conditioning sessions, the mice received a chocolate-flavored snack at the end of the corridor. During the test session, the shock was excluded. In addition, the moment when the mouse’s whole body emerged out of the shelter (seeking initiation) was labeled.

#### Predicted gain test 2

Fasted mice received chocolate-flavored snacks in the L-shaped chamber (60 cm by 8.5 cm) during each trial. The moment when the mice made physical contact with the food was analyzed.

#### Predicted loss test 1

Fasted mice received a chocolate-flavored snack at the edge of an L-shaped chamber (60 cm by 8.5 cm) with a shelter (6 cm by 12 cm by 18 cm triangle box). During conditioning sessions (3 days), each trial started when a door was removed (“accessibility moment”) with scheduled timing from the experimenter. During the test sessions, the mice were either chased or pushed by force into the shelter by the experimenter after receiving a chocolate-flavored snack. The door was reinstalled in front of the entrance of the shelter to deprive accessibility to the chocolate-flavored snacks.

#### Predicted loss test 2

A chocolate-flavored snack was placed in the tray on one side of the wall. During conditioning sessions, the fasted mice consumed the food in the tray, placed at an obtainable height of 8 cm. During test sessions, the fasted mice initiated eating behavior, rearing toward the visible food; however, the mice eventually abandoned eating behaviors when they realized that the food tray had an unobtainable height of 11 cm. The moment the mice voluntarily abandoned eating behaviors to the hanging food was labeled.

#### Predicted loss test 3

A food cue (vertical stripe) and a no-food cue (horizontal stripe) were randomly presented at the edge of an L-shaped chamber. During conditioning, the mice received chocolate-flavored snacks only when the food cue was presented. The success rate was recorded during training until it reached 80%, as reported previously (*38*). During the experiment, although the fasted mice initiated seeking after the food cue was presented, eventually, they abandoned eating voluntarily, on realizing that food was not available.

#### The multi-predicted gain test

Conditioning sessions (days 1 and 2) were performed for 15 trials each day to provide sufficient experience to the mice for learning about the location of a chocolate-flavored snack. The test session (day 3) was also performed for 15 trials. Each trial started when a door was removed (accessibility moment) with the scheduled timing from the experimenter. “Seeking initiation” was defined when the mice started emerging out of the shelter. “Proximate to food” was defined when the mice arrived at the top of the bridge. “Food contact” was defined as the moment when the mice came in physical contact with the food.

#### The multi-predicted gain/loss test

The fasted mice received a chocolate-flavored snack in the same chamber of the multi-predicted gain test. The experimenter closed the door after the end of the multi-gain events to measure neural activity predicted loss when “inaccessibility” occurred. “Consumption end” was labeled when the mice stopped contact with food in the hand. Otherwise, consumption end was labeled when the mice started to turn their head away from where the food was.

#### Eating-evoking test

Ad libitum mice were caged in a small cylindrical structure (10 cm diameter by 15 cm height) containing three large palatable cheese-flavored snacks and habituated for 5 min before laser stimulation. When the mice were proximate to food (i.e., when the mice were indisputably headed toward food), they were randomly given laser stimulation for 10 s. The LH^LepR^ neurons were stimulated for 10 s after a 1-min interval between each trial, and the AgRP neurons were given a wash-out period of at least 1 hour after each trial to minimize the sustained effect of the feeding behavior (*44*).

### Neural activity model

#### Preprocessing of data and kernel for model fitting

In this study, the preprocessing included aligning the fluorometric data to the first event point, performing *z*-scoring, conditional averaging, and up-sampling the data to 100 Hz. Single-trial analysis was performed similarly but without averaging across trials.

To predict photometric data from the predicted latent variable, a single-pulse response filter (kernel) was used (*58*). In addition, an offset was introduced to address the effect of negative values in the *z*-scored signal. A sharp increase occurred at the beginning of the predicted data due to the convolution operation using the GCamp6s kernel. For this reason, in the multi-predicted gain test, we used the last 5 s of the 10-s initial inaccessibility duration. For all other tasks, we added the first 125 samples before trial start with the average value of the first 50 samples after start.

#### Model-fitting procedure

Need and motivation models predict activity of neurons based on the specific events of each experiment. We chose different time windows for each event in the experiment to prevent interference of another event. For predicted gain test 1, a seeking moment was selected with a time window of 3 s as mice took about 3 s to show proximity to food event after seeking moment. Such interferences were not considered in other tests as the event being analyzed was the last event. Furthermore, the time window was selected to show sufficient amounts of increase or decrease following the predicted gain or loss for visualization. The fitting equations used for each experiment are explained in the following sections, from which the latent values were obtained and convolved with the GCaMP6s kernel to predict the empirical data. The predicted values were then compared to the actual data to obtain the optimal set of parameters with the lowest root mean square error (RMSE). In the single-trial analysis, we used neural response and predictions from events that vary across trials. Afterward, latent values were obtained as described above to be convolved with GCaMP6s kernel to predict the empirical data. For the individual trial analysis (multi-predicted gain/loss model), we divided the data into training and test sets using leave-one-out cross-validation (LOO-CV), and the same set of parameters was used to minimize RMSE for each experimental session.

An initial set of 100 parameters was randomly provided for executing the aforementioned process. MATLAB’s fmincon function was used to find the optimal parameters with the lowest RMSE. Last, the Akaike information criterion (AIC) (*59*) was obtained to compare the performance of models and determine which model best explained the neural activity

$$\mathrm{AIC}=N\times \text{ln}\;\left(\frac{\mathrm{RSS}}{N}\right)+2K$$

*N* is the length of the data, *RSS* is the squared sum of the residuals, and *K* is the number of free parameters used in the model fitting. We calculated the AIC for each model. When comparing the models, the model with the lower *AIC* that explained the data better was chosen. In addition, statistical tests were performed with *AIC* values.

#### Predicted gain test 1,2 and multi-predicted gain test

Predicted gain test 1,2 and multi-predicted gain test have the following sequence of events that induce predicted gain (Figs. 2, F and N, and 3A). On the basis of each event for each test, the parameters of each model are set to fit each model. We implemented the need model using the following equation

$$N\left(t\right)=N\left(t_{0}\right)-R\left(t\right)$$ (1)

We assumed that the activity of neurons encoding need decreases in a stepwise manner as the animal goes through a series of events (eqs. S17 and S18). We coded the need-encoding neurons with the structure of Eq. 1. *N*(*t*) is the value for need at the time *t*, and *N*(*t*_0_) is the value of need at the beginning of the experiment. *R*(*t*) indicates the stepwise increase of predicted gain, signaled by experimental events. *R*(*t*) is positive in these gain tasks

$$M\left(t\right)=\int_{t_{0}}^{t}a\times N\left(t\right)-\text{Leak}\;\mathrm{dt}$$ (2)

As the animal goes through a series of events, we assumed that the activity of the neurons that code for motivation is the integral of the state of need from the initial time to that point multiplied by the parameter “*a*” minus the value associated with “*Leak*.” Leak was defined as a constant rate by which the motivation (accumulated need) gradually reduces. Without Leak, once motivation is formed, it would not vanish over time. Since *a* and *Leak* are fixed as constant for each event, and the value of need is also fixed at a constant value, the neurons that code for motivation can be coded as a linear function over each interval. We coded motivation with the structure of Eq. 2.

(a) Comparison with the model for individual event models. The equations used in the models are the same as the equations above, but the change in *R*(*t*) varies only once in the individual event model.

(b) Multi-predicted gain/loss model. In the multi-predicted gain model, we additionally analyzed changes in need and motivational state after the mice had finished eating (Fig. 4). In this case, because of variability in the timing of food consumption in each trial, averaging by condition was not possible and individual trial analyzes were performed (fig. S5). The trials were divided into training and test sets using LOO-CV. Best-fit parameters were obtained by minimizing RMSE within the test set. Example traces (Fig. 4, C and E, and fig. S5, A and B) and goodness-of-fit metrics such as AIC were obtained in the test set. The prediction of each model during the event that induced predicted gain/loss was the same as the previous equations. In the event that induced predicted loss, *R*(*t*) was a negative value (Eqs. 1 and 2). To account for the case where the observer’s measurement of when the mouse shows that consumption end does not accurately pinpoint the actual time when the consummatory behavior stops because of food remaining in the mouth, we additionally fit the model with a 0 to 8 s of time delay (multi-predicted gain/loss; figs. S5 and S6).

#### Predicted loss test 1

Predicted loss test 1 proceeded as follows: (Fig. 3A) In this test, only the event that induced predicted loss (inaccessible) exists. On the basis of the equations described above (Eqs. 1 and 2), we implemented the code.

#### Predicted loss test 2,3

Predicted loss test 2 has the following sequence of events (Fig. 3I). For each event, we set the parameters to match each model. Unlike the predicted gain tests, in predicted loss test 2,3 (Fig. 3 and fig. S2), the prediction was constrained to return to the baseline value before the trial start when the event that induced predicted loss was given (eating abandon). In this event, *R*(*t*) was 0. The equation of each model was described above (Eqs. 1 and 2).

### Behavior activity model

#### Preprocessing of data

All data were analyzed using MATLAB 2022B or stated otherwise. A probability of behavioral activity was calculated from periodic mouse behavior annotations by dividing a 1-s time period into bins: 10 bins for the eating-evoking test (Fig. 5). Whenever a behavior occurred during a bin, the bin was recorded as “1.” Subsequently, all time bins in a test per animal were averaged and normalized to derive a probability of behavioral activity. For each time bin, the behavioral activity was calculated as a moving average (10 bins for the 10-s stimulus test and 60 bins for the other bins) to create a smooth curve for model comparison.

#### Model-fitting procedure

The need model defined that upon neuron activation at time *t_s On_*, the need state exhibited a value higher than its initial value. This higher need was continuously integrated to calculate the motivation level *BN*(*t*), which was normalized according to the maximum value of the predicted data. *A* is the slope created when the stimulus-induced increase in need is integrated over time until time *t_s Off_*. The motivation level was also calculated as a leaky integrated model based on the decrease in the need during the stimulus offset, such that the maximum decrease rate was not above one-fourth of the increase rate during the stimulus on.

This calculated motivation level generated a behavioral activity when crossing the threshold (generated behavior threshold). In addition, for the generated behavior, the probability of the behavior remained at a value of 1 because the motivation level continued to maintain the behavior above a certain threshold (maintained behavior threshold). Subsequently, the motivation level gradually decreased when the stimulus disappeared and only the effect of the intact *Leak* remained. In addition, the probability of action decreased when the motivation level fell below the threshold (maintained behavior threshold).

For the motivation model, the motivation level *BM*(*t*) was observed to sharply rise (*A_stim_*) at the stimulus onset (*t_s On_*). The motivation level remained constant until the stimulus offset (*t_s Off_*), after which it returned to the initial level.

In addition, the code was implemented to compute the delay (*t_delay_*) between the actual neural activity and the behavioral results induced by the optogenetic stimulus and the threshold (generated behavior threshold) using fmincon function in MATLAB for the best-fit parameters with the smallest RMSE (Eqs. 3, 4). Since the probability of an animal’s behavior was modeled, the boundary values were set from 0 to 1. In the behavioral probability model, the threshold (maintained behavior threshold) was set to 1, which meant that values greater than 1 were fixed at 1. In the model, the behavioral probability value was 1, but the state related to motivation was constantly increasing when the stimulus was on, and slowly decreasing when the stimulus was off because of a *Leak*. Similar to the process of the neural activity model, AICs were obtained from the predicted behavior model and actual behavior, and a statistical test was performed using the obtained AICs

##### (i) Need

$$\mathrm{BN}\left(t\right)=\left\{ \begin{array}{c} {\mathrm{BN}}_{0}\left(t_{0}\leq t<t_{s\;\mathrm{On}}+t_{\text{delay}}\right)\\ {\mathrm{BN}}_{0}+A\times \left(t-t_{s\;\mathrm{On}}\right)\;\left(t_{s\;\mathrm{On}}+t_{\text{delay}}\leq t<t_{s\;\mathrm{Off}}+t_{\mathrm{de}l\mathrm{ay}}\right)\\ {\mathrm{BN}}_{0}+A\times \left(t_{s\;\mathrm{Off}}-t_{s\;\mathrm{On}}\right)-\text{Leaky}\;\left(t-t_{s\;\mathrm{On}}\right)\;\left(t_{s\;\mathrm{Off}}+t_{\text{delay}}\leq t\right) \end{array}\right.$$ (3)

[If BN(t)≥1,BN(t)=1, else,if BN(t)≤0,BN(t)=0]

##### (ii) Motivation

$$\mathrm{BM}\left(t\right)=\left\{ \begin{array}{c} {\mathrm{BM}}_{0}\;\left(t_{0}\leq t<t_{s\;\mathrm{On}}+t_{\text{delay}}\right)\\ {\mathrm{BM}}_{0}+A_{\text{stim}}\;\left(t_{s\;\mathrm{On}}+t_{\text{delay}}\leq t<t_{s\;\mathrm{Off}}+t_{\text{delay}}\right)\\ {\mathrm{BM}}_{0}\;\left(t_{s\;\mathrm{Off}}+t_{\text{delay}}\leq t\right) \end{array}\right.$$ (4)

[If BN(t)≥1,BN(t)=1,else,if BN(t)≤0,BN(t)=0]

#### Simulation procedure

We examined different strategies of an agent using a computer simulation when the dynamics of need follows our normative framework. In the simulated environment, the agent forages for food resources over a long distance, experiencing a progressive increase in the deficit over time. This ensures that the go/no-go decision-making process during foraging is induced by ecological factors that align with natural conditions. We tested the necessity of accumulation using two different simulation conditions.

##### Need model (direct need)

Actions were generated when the need value surpassed a threshold, and actions ceased when the need fell below the threshold.

(i) Reward prediction changes when the agent stops. In this situation, the agent can predict a decreasing deficit in the future while it is moving. However, if the agent stops, the agent immediately updates the prediction based on the increased future deficit.

(ii) Reward prediction is not affected by the motion of the agent. In this situation, even if the agent stops, it computes a decreasing deficit due to the reward it will eat later.

##### Motivation model (accumulated need)

Actions were initiated when the motivation, the accumulation of need, exceeded a threshold. Actions were halted when the motivation value fell below the threshold.

(i) Always accessible state condition.

The agent starts with the accessible condition. In these conditions the initial deficit at time 0 was assigned a value of 1 with subsequent increments of 0.00001 per time step.

(ii) With inaccessible state condition.

The agent starts in an initial state where the deficit remains at zero for 100 time steps, after which an inaccessible state is introduced, causing the deficit to gradually increase over the next 500 time steps. During this period of inaccessibility, the deficit increases linearly until it reaches a value of 1. Furthermore, in this condition, we conducted two simulations based on whether the deficit increases in the accessible state. This condition closely mimics the actual experimental setup without introducing any additional events beyond accessible events in the experimental environment. In contrast to other simulations, we track the agent for 1000 time steps, starting from the moment the food is consumed, to illustrate the post-food consumption process. The deficit starts to decrease when the food is eaten by 0.012 per time step and never falls below zero.

a) Increasing deficit during accessible condition (ID) increases the agent’s deficit by 0.00001 in the accessible state.

b) Not increasing deficit during accessible condition (NID), during the accessible state, the deficit remains constant except for random noise.

(iii) Simulation settings.

(a) The basis of simulation: The agent was modeled on the basis of model rationality (eqs. S1~S15).

(b) Model compute: For the computation of sum of discounted deficit (SDD), r was defined as 0.99 (Eq. 3). For the computation of predicted event (PE), the parameter “e” was specified as 0.1 (Eq. 4), while the accumulation parameter “a” for calculating motivation was set as 0.0015 (eq. S10). As motivation is calculated through the accumulation of need, it is set to decrease by 0.001 per 1 time step for *Leak*. In the NID condition, *Leak* parameter was set 0.0008. For need value, the process was to treat values below 0 as 0.

(c) Action generation: The threshold for generating the behavior was defined as 0.5. We also considered the delay due to the behavioral switch between stay and go actions (*60*). The agent was set to maintain the stop behavior at least 50 time steps after the switch, and for the go behavior, it is set to hold for 10 times steps.

(d) The model parameters were systematically explored across a wide range to ensure robustness and reliability of our simulations. Specifically, thresholds and integrated effect factors were varied between 0.1 and 1 to investigate their impact on model performance. In addition, the discounting factor, ranging from 0.9 to 1, was manipulated to gauge its influence on future consideration within the model. This comprehensive parameter exploration allowed for a thorough understanding of how variations in model parameters affect simulated behaviors and outcomes, contributing to the robustness and generalizability of our findings.

### Model permutation test procedure

To verify our model trace fit with experimental data rather than random data, raw calcium traces or behavior activity were shuffled into a random dataset. Afterward, the residual sum of squares was calculated for the model-random dataset and the model-experimental dataset. Afterward, the permutation test was permuted 1000 times between the two datasets.

### PCA analysis

For preprocessing, neural activity was collected from the multi-predicted gain test trials from AgRP or LH^LepR^ neurons. All the trials were subtracted with its corresponding average value from the whole trial and concatenated to a matrix (3000 frames per trial) to be processed with MATLAB function principal components analysis (PCA). The product of principal component and individual neural activity (coeff) was used to make the neural trajectory for analysis. PC1, PC2, and PC3 explained about 80.9, 9.2, 3.6% of the variance, respectively. Afterward, PC1 data were processed with MATLAB’s function tsne with perplexity 10 for t-SNE distribution results.

### CEBRA analysis

Since PCA analysis is a linear combination of the original data, we used CEBRA (*61*), a nonlinear analysis method, to uncover neural dynamics in a high-performance latent space to reveal potential hidden latent variables. We fitted the AgRP or LH^LepR^ neuronal data from multi-prediction gain tests with continuously labeled time frames in predicted gain events. For preprocessing, neural activity was collected from the multi-predicted gain test trials from AgRP or LH^LepR^ neurons. The trials were reshaped to a matrix form comprising three trials (750 frames per trial) × *n* dimension (*n* for AgRP = 24, *n* for LH^LepR^ = 19). Afterward, the neural activity was up-sampled to 100 Hz/s (total 3000 frames). The behavior label was differentiated to continuous label scores for all-time frames to match the number of columns of the reshaped trial matrix: trial start = 0 (0 s)/accessibility moment = 1/seeking moment = 2/proximate to food = 3/contact = 4/trial end = 5 (the 30 s). After preprocessing, 70% of the trial matrix and continuous behavior labels were split for the training set, and the rest were designated test sets. Continuous behavior labels were then shuffled to make shuffled sets for model fitting. The preprocessed datasets were used in the CEBRA modeling function (Python 3) (*61*) that was built with the following parameters: learning rate, 0.00005; maximum iteration, 100,000; temperature_mode, auto; model_architecture, “offset10_model”; batch_size, 1024, outpud_dimension = 3; time_offsets = 10; conditional = “time_delta.” The plot of temperature change and loss was used with functions cebra.plot_loss and cebra.plot_temperature. Consistency scores between hypothalamic neurons were computed using the consistency_score function. Behavior prediction was decoded from embeddings using k-nearest neighbor decoder. The prediction was used with real labels to compute RMSE for comparison between original and shuffled data.

### Cross-validation analysis

In this study, we applied LOO-CV to evaluate the performance of a single-trial fitting model. This process is repeated for each trial in the dataset, ensuring that every trial is used once as a test case. We used the parameter set derived from training sets with the lowest RMSE to calculate the test set’s AIC. Then, the AIC results from all test sets were averaged to obtain an AIC from an individual animal (data points in Fig. 4, D and F, and fig. S6, B and D).

### Statistical tests

Statistical tests were performed by Friedman’s test with each model’s AIC value. The Friedman test is a nonparametric statistical method used to compare differences of observations based on rank orders (*62*). A permutation test was performed if statistical significance was not secured in Friedman’s test because of insufficient data. The raw data were divided into 20 parts, and each part was sampled without replacement to generate 1000 sampling data of the same size as the raw data (fig. S2, E to G). Subsequently, the model-fitting process was performed on the sampled data to obtain the AIC distribution of each model. Furthermore, this AIC distribution and the AIC of the actual data were used for obtaining the empirical *P* value (fig. S2, F and G).

## Supplementary Materials

### The PDF file includes:

Supplementary Text Figs. S1 to S9 Tables S1 and S2 Legends for movies S1 to S5 References

## References

[1] W. B. Cannon, *The Wisdom of the Body* (W W Norton & Co, 1932), pp. 312–312.

[2] W. E. Allen, L. A. DeNardo, M. Z. Chen, C. D. Liu, K. M. Loh, L. E. Fenno, C. Ramakrishnan, K. Deisseroth, L. Luo, Thirst-associated preoptic neurons encode an aversive motivational drive. Science 357, 1149–1155 (2017). 28912243 10.1126/science.aan6747 PMC5723384

[3] Y. H. Lee, M. Kim, M. Lee, D. Shin, D. S. Ha, J. S. Park, Y. B. Kim, H. J. Choi, Food craving, seeking, and consumption behaviors: Conceptual phases and assessment methods used in animal and human studies. J. Obes. Metab. Syndr. 28, 148–157 (2019). 31583379 10.7570/jomes.2019.28.3.148 PMC6774451

[4] G. Pezzulo, F. Rigoli, K. Friston, Active Inference, homeostatic regulation and adaptive behavioural control. Prog. Neurobiol. 134, 17–35 (2015). 26365173 10.1016/j.pneurobio.2015.09.001 PMC4779150

[5] K. Juechems, J. Balaguer, S. Herce Castañón, M. Ruz, J. X. O’Reilly, C. Summerfield, A network for computing value equilibrium in the human medial prefrontal cortex. Neuron 101, 977–987.e3 (2019). 30683546 10.1016/j.neuron.2018.12.029

[6] C. L. Hull, *Principles of Behavior: An Introduction to Behavior Theory* (Appleton-Century, 1943), pp. 422.

[7] K. C. Berridge, Motivation concepts in behavioral neuroscience. Physiol. Behav. 81, 179–209 (2004). 15159167 10.1016/j.physbeh.2004.02.004

[8] J. Bosulu, G. Pezzulo, S. Hétu, A computational account of needing and wanting. bioRxiv 513547 [Preprint] (2023). 10.1101/2022.10.24.513547.

[9] O. J. Hulme, T. Morville, B. Gutkin, Neurocomputational theories of homeostatic control. Phys. Life Rev. 31, 214–232 (2019). 31395433 10.1016/j.plrev.2019.07.005

[10] H. R. Kim, A. N. Malik, J. G. Mikhael, P. Bech, I. Tsutsui-Kimura, F. Sun, Y. Zhang, Y. Li, M. Watabe-Uchida, S. J. Gershman, N. Uchida, A unified framework for dopamine signals across timescales. Cell 183, 1600–1616.e25 (2020). 33248024 10.1016/j.cell.2020.11.013 PMC7736562

[11] J. Cox, A. R. Minerva, W. T. Fleming, C. A. Zimmerman, C. Hayes, S. Zorowitz, A. Bandi, S. Ornelas, B. McMannon, N. F. Parker, I. B. Witten, A neural substrate of sex-dependent modulation of motivation. Nat. Neurosci. 26, 274–284 (2023). 36646878 10.1038/s41593-022-01229-9 PMC12232987

[12] K. Choi, E. Piasini, E. Díaz-Hernández, L. V. Cifuentes, N. T. Henderson, E. N. Holly, M. Subramaniyan, C. R. Gerfen, M. V. Fuccillo, Distributed processing for value-based choice by prelimbic circuits targeting anterior-posterior dorsal striatal subregions in male mice. Nat. Commun. 14, 1920 (2023). 37024449 10.1038/s41467-023-36795-4 PMC10079960

[13] M. Keramati, B. Gutkin, Homeostatic reinforcement learning for integrating reward collection and physiological stability. eLife 3, e04811 (2014). 25457346 10.7554/eLife.04811 PMC4270100

[14] C. Gizowski, C. Zaelzer, C. W. Bourque, Clock-driven vasopressin neurotransmission mediates anticipatory thirst prior to sleep. Nature 537, 685–688 (2016). 27680940 10.1038/nature19756

[15] C. Deans, Biological prescience: The role of anticipation in organismal processes. Front. Physiol. 12, 672457 (2021). 34975512 10.3389/fphys.2021.672457 PMC8719636

[16] V. Augustine, S. K. Gokce, Y. Oka, Peripheral and central nutrient sensing underlying appetite regulation. Trends Neurosci. 41, 526–539 (2018). 29914721 10.1016/j.tins.2018.05.003 PMC6064385

[17] Y. Livneh, A. U. Sugden, J. C. Madara, R. A. Essner, V. I. Flores, L. A. Sugden, J. M. Resch, B. B. Lowell, M. L. Andermann, Estimation of current and future physiological states in insular cortex. Neuron 105, 1094–1111.e10 (2020). 31955944 10.1016/j.neuron.2019.12.027 PMC7083695

[18] F. Reed, S. H. Lockie, A. Reichenbach, C. J. Foldi, Z. B. Andrews, Appetite to learn: An allostatic role for AgRP neurons in the maintenance of energy balance. Curr. Opin. Endocr. Metab. Res. 24, 100337 (2022).

[19] M. Torigoe, T. Islam, H. Kakinuma, C. C. A. Fung, T. Isomura, H. Shimazaki, T. Aoki, T. Fukai, H. Okamoto, Zebrafish capable of generating future state prediction error show improved active avoidance behavior in virtual reality. Nat. Commun. 12, 5712 (2021). 34588436 10.1038/s41467-021-26010-7 PMC8481257

[20] C. A. Zimmerman, Y. C. Lin, D. E. Leib, L. Guo, E. L. Huey, G. E. Daly, Y. Chen, Z. A. Knight, Thirst neurons anticipate the homeostatic consequences of eating and drinking. Nature 537, 680–684 (2016). 27487211 10.1038/nature18950 PMC5161740

[21] D. S. Ramsay, S. C. Woods, Clarifying the roles of homeostasis and allostasis in physiological regulation. Psychol. Rev. 121, 225–247 (2014). 24730599 10.1037/a0035942 PMC4166604

[22] C. L. Chen, F. Aymanns, R. Minegishi, V. D. V. Matsuda, N. Talabot, S. Günel, B. J. Dickson, P. Ramdya, Ascending neurons convey behavioral state to integrative sensory and action selection brain regions. Nat. Neurosci. 26, 682–695 (2023). 36959417 10.1038/s41593-023-01281-z PMC10076225

[23] T. Akam, I. Rodrigues-Vaz, I. Marcelo, X. Zhang, M. Pereira, R. F. Oliveira, P. Dayan, R. M. Costa, The anterior cingulate cortex predicts future states to mediate model-based action selection. Neuron 109, 149–163.e7 (2021). 33152266 10.1016/j.neuron.2020.10.013 PMC7837117

[24] *The Interoceptive Mind: From Homeostasis to Awareness*, M. Tsakiris, H. De Preester Eds. (Oxford Univ. Press, 2018).

[25] B. S. McEwen, J. C. Wingfield, The concept of allostasis in biology and biomedicine. Horm. Behav. 43, 2–15 (2003). 12614627 10.1016/s0018-506x(02)00024-7

[26] R. A. Hinde, Ethological models and the concept of ’drive‘. Br. J. Philos. Sci. 6, 321–331 (1956).

[27] D. S. Lehrman, A critique of Konrad Lorenz’s theory of instinctive behavior. Q. Rev. Biol. 28, 337–363 (1953). 13121237 10.1086/399858

[28] R. Dawkins, A threshold model of choice behaviour. Anim. Behav. 17, 120–133 (1969).

[29] K. Z. Lorenz, The comparative method in studying innate behavior patterns, in *Physiological Mechanisms in Animal Behavior. (Society's Symposium IV.)* (Academic Press, 1950), pp. 221–268.

[30] J. D. Davidson, A. El Hady, Foraging as an evidence accumulation process. PLOS Comput. Biol. 15, e1007060 (2019). 31339878 10.1371/journal.pcbi.1007060 PMC6682163

[31] D. O. Hebb, *The Organization of Behavior: A Neuropsychological Theory* (Wiley, 1949), pp. 335.

[32] S. Luquet, F. A. Perez, T. S. Hnasko, R. D. Palmiter, NPY/AgRP neurons are essential for feeding in adult mice but can be ablated in neonates. Science 310, 683–685 (2005). 16254186 10.1126/science.1115524

[33] Q. Gao, T. L. Horvath, Neurobiology of feeding and energy expenditure. Annu. Rev. Neurosci. 30, 367–398 (2007). 17506645 10.1146/annurev.neuro.30.051606.094324

[34] T. M. Hahn, J. F. Breininger, D. G. Baskin, M. W. Schwartz, Coexpression of Agrp and NPY in fasting-activated hypothalamic neurons. Nat. Neurosci. 1, 271–272 (1998). 10195157 10.1038/1082

[35] J. D. Deem, C. L. Faber, G. J. Morton, AgRP neurons: Regulators of feeding, energy expenditure, and behavior. FEBS J. 289, 2362–2381 (2022). 34469623 10.1111/febs.16176 PMC9040143

[36] I. C. Alcantara, A. P. M. Tapia, Y. Aponte, M. J. Krashes, Acts of appetite: Neural circuits governing the appetitive, consummatory, and terminating phases of feeding. Nat. Metab. 4, 836–847 (2022). 35879462 10.1038/s42255-022-00611-y PMC10852214

[37] Q. Liu, X. Yang, M. Luo, J. Su, J. Zhong, X. Li, R. H. M. Chan, L. Wang, An iterative neural processing sequence orchestrates feeding. Neuron 111, 1651–1665.e5 (2023). 36924773 10.1016/j.neuron.2023.02.025

[38] Y. H. Lee, Y. B. Kim, K. S. Kim, M. Jang, H. Y. Song, S. H. Jung, D. S. Ha, J. S. Park, J. Lee, K. M. Kim, D. H. Cheon, I. Baek, M. G. Shin, E. J. Lee, S. J. Kim, H. J. Choi, Lateral hypothalamic leptin receptor neurons drive hunger-gated food-seeking and consummatory behaviours in male mice. Nat. Commun. 14, 1486 (2023). 36932069 10.1038/s41467-023-37044-4 PMC10023672

[39] S. Shin, I. J. You, M. Jeong, Y. Bae, X. Y. Wang, M. L. Cawley, A. Han, B. K. Lim, Early adversity promotes binge-like eating habits by remodeling a leptin-responsive lateral hypothalamus-brainstem pathway. Nat. Neurosci. 26, 79–91 (2023). 36510113 10.1038/s41593-022-01208-0 PMC9829538

[40] M. E. Mazurek, J. D. Roitman, J. Ditterich, M. N. Shadlen, A role for neural integrators in perceptual decision making. Cereb. Cortex 13, 1257–1269 (2003). 14576217 10.1093/cercor/bhg097

[41] S. Bitzer, H. Park, F. Blankenburg, S. J. Kiebel, Perceptual decision making: Drift-diffusion model is equivalent to a Bayesian model. Front. Hum. Neurosci. 8, 102 (2014). 24616689 10.3389/fnhum.2014.00102 PMC3935359

[42] O. B. Artun, H. Z. Shouval, L. N. Cooper, The effect of dynamic synapses on spatiotemporal receptive fields in visual cortex. Proc. Natl. Acad. Sci. U.S.A. 95, 11999–12003 (1998). 9751779 10.1073/pnas.95.20.11999 PMC21754

[43] D. Millman, S. Mihalas, A. Kirkwood, E. Niebur, Self-organized criticality occurs in non-conservative neuronal networks during Up states. Nat. Phys. 6, 801–805 (2010). 21804861 10.1038/nphys1757 PMC3145974

[44] Y. Chen, R. A. Essner, S. Kosar, O. H. Miller, Y. C. Lin, S. Mesgarzadeh, Z. A. Knight, Sustained NPY signaling enables AgRP neurons to drive feeding. eLife 8, e46348 (2019). 31033437 10.7554/eLife.46348 PMC6513552

[45] S. Rutherford, S. M. Kia, T. Wolfers, C. Fraza, M. Zabihi, R. Dinga, P. Berthet, A. Worker, S. Verdi, H. G. Ruhe, C. F. Beckmann, A. F. Marquand, The normative modeling framework for computational psychiatry. Nat. Protoc. 17, 1711–1734 (2022). 35650452 10.1101/2021.08.08.455583 PMC7613648

[46] E. B. Richman, N. Ticea, W. E. Allen, K. Deisseroth, L. Luo, Neural landscape diffusion resolves conflicts between needs across time. Nature 623, 571–579 (2023). 37938783 10.1038/s41586-023-06715-z PMC10651489

[47] T. T. Hills, P. M. Todd, D. Lazer, A. D. Redish, I. D. Couzin, Exploration versus exploitation in space, mind, and society. Trends Cogn. Sci. 19, 46–54 (2015). 25487706 10.1016/j.tics.2014.10.004 PMC4410143

[48] A. Dickinson, Actions and habits: The development of behavioural autonomy. Philos. Trans. R. Soc. Lond. B Biol. Sci. 308, 67–78 (1985).

[49] H. H. Yin, B. J. Knowlton, B. W. Balleine, Lesions of dorsolateral striatum preserve outcome expectancy but disrupt habit formation in instrumental learning. Eur. J. Neurosci. 19, 181–189 (2004). 14750976 10.1111/j.1460-9568.2004.03095.x

[50] N. D. Daw, J. P. O’Doherty, Chapter 21 - Multiple systems for value learning, in *Neuroeconomics (Second Edition)*, P. W. Glimcher, E. Fehr, Eds., (Academic Press, 2014), pp. 393–410.

[51] N. M. Seel, Model-based learning: A synthesis of theory and research. Educ. Technol. Res. Dev. 65, 931–966 (2017).

[52] Y. Chen, Y. C. Lin, T. W. Kuo, Z. A. Knight, Sensory detection of food rapidly modulates arcuate feeding circuits. Cell 160, 829–841 (2015). 25703096 10.1016/j.cell.2015.01.033 PMC4373539

[53] A. Petzold, H. E. van den Munkhof, R. Figge-Schlensok, T. Korotkova, Complementary lateral hypothalamic populations resist hunger pressure to balance nutritional and social needs. Cell Metab. 35, 456–471.e6 (2023). 36827985 10.1016/j.cmet.2023.02.008 PMC10028225

[54] M. R. Zimmer, A. H. O. Fonseca, O. Iyilikci, R. D. Pra, M. O. Dietrich, Functional ontogeny of hypothalamic Agrp neurons in neonatal mouse behaviors. Cell 178, 44–59.e7 (2019). 31104844 10.1016/j.cell.2019.04.026 PMC6688755

[55] Y. Aponte, D. Atasoy, S. M. Sternson, AGRP neurons are sufficient to orchestrate feeding behavior rapidly and without training. Nat. Neurosci. 14, 351–355 (2011). 21209617 10.1038/nn.2739 PMC3049940

[56] J. N. Siemian, M. A. Arenivar, S. Sarsfield, C. B. Borja, C. N. Russell, Y. Aponte, Lateral hypothalamic LEPR neurons drive appetitive but not consummatory behaviors. Cell Rep. 36, 109615 (2021). 34433027 10.1016/j.celrep.2021.109615 PMC8423025

[57] I. Aklan, N. Sayar Atasoy, Y. Yavuz, T. Ates, I. Coban, F. Koksalar, G. Filiz, I. C. Topcu, M. Oncul, P. Dilsiz, U. Cebecioglu, M. I. Alp, B. Yilmaz, D. R. Davis, K. Hajdukiewicz, K. Saito, W. Konopka, H. Cui, D. Atasoy, NTS catecholamine neurons mediate hypoglycemic hunger via medial hypothalamic feeding pathways. Cell Metab. 31, 313–326.e5 (2020). 31839488 10.1016/j.cmet.2019.11.016 PMC9017597

[58] T. W. Chen, T. J. Wardill, Y. Sun, S. R. Pulver, S. L. Renninger, A. Baohan, E. R. Schreiter, R. A. Kerr, M. B. Orger, V. Jayaraman, L. L. Looger, K. Svoboda, D. S. Kim, Ultrasensitive fluorescent proteins for imaging neuronal activity. Nature 499, 295–300 (2013). 23868258 10.1038/nature12354 PMC3777791

[59] H. Akaike, A new look at the statistical model identification. IEEE Trans. Automat. Contr. 19, 716–723 (1974).

[60] J. J. Orban de Xivry, P. Lefèvre, A switching cost for motor planning. J. Neurophysiol. 116, 2857–2868 (2016). 27655964 10.1152/jn.00319.2016 PMC5168003

[61] S. Schneider, J. H. Lee, M. W. Mathis, Learnable latent embeddings for joint behavioural and neural analysis. Nature 617, 360–368 (2023). 37138088 10.1038/s41586-023-06031-6 PMC10172131

[62] M. Friedman, The use of ranks to avoid the assumption of normality implicit in the analysis of variance. J. Am. Stat. Assoc. 32, 675–701 (1937).

[63] F. M. Toates, Homeostasis and drinking. Behav. Brain Sci. 2, 95–102 (1979).

[64] A. K. Seth, Interoceptive inference, emotion, and the embodied self. Trends Cogn. Sci. 17, 565–573 (2013). 24126130 10.1016/j.tics.2013.09.007

[65] M. A. Apps, M. Tsakiris, The free-energy self: A predictive coding account of self-recognition. Neurosci. Biobehav. Rev. 41, 85–97 (2014). 23416066 10.1016/j.neubiorev.2013.01.029 PMC3848896

[66] M. Hovd, R. R. Bitmead, Feedforward for stabilization in the presence of constraints. J. Process Control 22, 659–665 (2012).

[67] K. C. Berridge, Separating desire from prediction of outcome value. Trends Cogn. Sci. 27, 932–946 (2023). 37543439 10.1016/j.tics.2023.07.007 PMC10527990

