# Supplementary material for: A normative framework dissociates need and motivation in hypothalamic neurons
Source: Sci Adv. 2024 Nov 6;10(45):eado1820. doi: 10.1126/sciadv.ado1820 (PMC11540019; doi:10.1126/sciadv.ado1820)
Supplement: Supplementary file 1 — Supplementary Text Figs. S1 to S9 Tables S1 and S2 Legends for movies S1 to S5 References [file sciadv.ado1820_sm.pdf]

Science Advances
AAAS

Supplementary Materials for

A normative framework dissociates need and motivation in hypothalamic neurons

Kyu Sik Kim *et al.*

Corresponding author: HyungGoo R. Kim, hyunggoo.r.kim@gmail.com; Hyung Jin Choi, hjchoi@snu.ac.kr

Sci. Adv. **10**, eado1820 (2024)
DOI: 10.1126/sciadv.ado1820

The PDF file includes:

Supplementary Text
Figs. S1 to S9
Tables S1 and S2
Legends for movies S1 to S5
References

Other Supplementary Material for this manuscript includes the following:

Movies S1 to S5

Supplementary Text

Theoretical Backgrounds

In the normative approach of homeostatic theory in all homeostatic contexts, individual contexts constitute independent axes in a multidimensional space H. An animal's deficit ($D_i(H_t)$) in homeostatic axis i and time t, is defined as the degree to which a state deviates from a homeostatic set point ($H^* = (h_1^*, h_2^*, ...)$) and homeostatic state($H_t = (h_{1,t}, h_{2,t}, ...)$) (*13*). Here, we redefine $D_i(H_t)$ as below (Equation S1). The newly defined $D_i(H_t)$ takes the difference between setpoint and state from each axis that affects homeostasis, and considers the impact of each factor (e.g., nonlinearities) independently through a function f_i. Then, the total deficit at time t ($D_{total}(H_t)$) is determined as the linear sum of the deficit from each axis (Equation S2).

$$D_i(H_t) = f_i\left(\left|h_i^* - h_{i,t}\right|\right) \text{ (Equation S1)}$$

$$D_{total}(H_t) = \sum_{i=1}^{N} f_i\left(\left|h_i^* - h_{i,t}\right|\right) = \sum_{i=1}^{N} D_i(H_t) \text{ (Equation S2)}$$

An internal state deviating from the homeostatic set point is perceived as a current deficit (*8*). The animal analyzes external information to predict future events, thereby forecasting its future predicted deficit in addition to its current deficit (*14-19*). The core task of the brain is to anticipate an animal's future deficits, regulating internal state for preparation of deficit changes. Therefore, for optimal survival, the predicted deficit should serve as the optimal update for change in state. The cumulative effect of $D_{total}(H_t)$ over time, as the state of homeostasis progresses along the trajectory (policy π), is defined as the sum of the discounted deficit in which γ is the discounting factor, and can also be understood as the sum of all discounted deficit ($SDD_\pi(H_t)$) (Equation S3).

$$SDD_\pi(H_t) = \sum_{j=t}^{n+t-1} \gamma^{j-t} \cdot D_{total}(H_{j+1}) \text{ (Equation S3)}$$

$SDD_\pi(H_t)$ is a value that predicts information about a future state based only on what is happening at the time t.

If the animal is given accessibility at time t_{acc}, the animal will try to minimize its deficit, and this process will cause a change in an animal's prediction (Fig. 1A). We define this amount as predicted change ($PC_{total}(H_t)$) (Equation S4). The predicted change at time t can be found by multiplying the difference between the sum of all discounted deficit at time t and time t_{acc} by a free parameter, w. The total predicted deficit ($PD_{total}(H_t)$) can be described as the sum of the total predicted deficit at the current state and the total predicted change (Fig. 1) (Equation S5).

$$PC_{total}(H_t) = w * [SDD_\pi(H_t) - SDD_\pi(H_{t_{acc}})] \text{ (Equation S4)}$$

$$PD_{total}(H_t) = D_{total}(H_t) + PC_{total}(H_t) \text{ (Equation S5)}$$

According to the normative approach in homeostatic theory, when animals expect a reward, PD decreases as PD includes future deficits (*13*). This reward can be expressed as the sum of discounted rewards ($SDR_\pi(H_t)$) by the state-value function (Equation S6). If the behavioral

policy that maximizes $SDR_\pi(H_t)$ aligns with the policy that minimizes $SDD_\pi(H_t)$, the animal can maintain homeostasis and learn through reinforcement, which is optimal for efficient survival (Equation S7,S8).

$$SDR_\pi(H_t) = \sum_{i=t}^{n+t-1} \gamma^{i-t} \cdot (r_i) \text{ (Equation S6)}$$

$$SDR_\pi(H_t) = D_{total}(H_t) + (\gamma - 1) \cdot SDD_\pi(H_t) \text{ (Equation S7)}$$

$$argmax(SDR_\pi(H_t)) = argmin(SDD_\pi(H_t)) \text{ (Equation S8)}$$

Since, according to our new definition of deficit (Equation S1,S2), $SDD_\pi(H_t)$ and $PD_{total}(H_t)$ are the linear sum of the differences along each homeostatic axis, the $PD_{total}(H_t)$ can also be expressed by linearity as the sum of the $PD_i(H_t)$ along each axis. When there are N homeostatic axes, the $PD_{total}(H_t)$ can be expressed as (Equation S9).

$$PD_{total}(H_t) = PD_1(H_t) + PD_2(H_t) + PD_3(H_t) + \cdots = \sum_{i=1}^{N} PD_i(H_t) \text{ (Equation S9)}$$

Minimizing the total predicted deficiency, $PD_{total}(H_t)$, would be important for the animal's survival. If a change is induced in only one axis (e.g., axis 1), such as in our controlled experiments, the policy that minimizes the $PD_1(H_t)$ on this one axis will be equivalent to the policy that minimizes the $PD_{total}(H_t)$. Also, these policies correspond to the ones that maximize the $SDR_\pi(H_t)$ (Equation S10).

$$argmin\left(PD_{total}(H_t)\right) = argmin\left(PD_1(H_t)\right) = argmax\left(SDR_\pi(H_t)\right) \text{ (Equation S10)}$$

Also, in controlled experiments with minimal food intake, the current deficit state has the same value at each time point as the trial progresses.

Note that the above relationship still holds in the previous study (*13*) in certain conditions. In the previous study, $D(H_t)$ was defined as follows:

$$D(H_t) = \sqrt[m]{\sum_{i=1}^{N} \left|h_i^* - h_{i,t}\right|^n} \text{ (Equation S11)}$$

When the free parameter m (Equation S7), which determines the $D(H_t)$, is set to 1, $D(H_t)$ can be expressed as the linear sum of homeostatic $D(H_t)$ on each axis of the factors influencing homeostasis. In this case, the $PD_{total}(H_t)$ can be divided by the $PD_i(H_t)$ of each homeostatic factor. Furthermore, in our controlled experiments, the homeostatic $D(H_t)$ may be mainly along a single context axis (i.e., food) that affects homeostasis (m=n=1; Equation S11). In such cases, PD can be considered as a factor that solely responds to variations of this homeostatic axis (food).

Within the realm of feeding, the current "food deficit" is $D_f(H_t)$ (Equation S1, fig. S1B Phase 2), while the changes in the sum of discounted future "food deficit" represents the sum of predicted changes (gain or loss) PC_f that alarm feedforward signals (Equation S4, fig. S1B Phase 3) to sum up to a predicted deficit for food ($PD_f(H_t)$) (Equation S12).

$$PD_f(H_t) = D_f(H_t) + PC_f(H_t) \text{ (Equation S12)}$$

It is known that the bodily system of an animal responds to the cue from the environment which might lead to homeostasis recovery to produce a predicted body state $PD_f(H_t)$ (*63-65*). This

anticipatory regulation system is paramount for survival because it generates real-time feedforward signals that prevents overcompensation of the bodily system when the bodily system changes back to the set point after state deviation (*16, 63, 66*). Consequently, in the hunger domain, $PD_f(H_t)$ should serve as the animal's optimal calculation for food "need" $N_f(t)$ (Equation S13).

$$PD_f(H_t) \rightarrow N_f(t) \text{ (Equation S13)}$$

In the domain of animal behavior and survival, animals constantly decide whether to seek for food or not, by combining complicated external cues and interoceptive states that are often uncertain. Animals often produce behaviors through accumulation of these cues (*4, 26, 27*). In perceptual decision making with noisy sensory evidence, lateral intraparietal area (LIP) neurons appear to accumulate sensory information (*40, 41*). The decision of leaving a patch or not can also be modeled with evidence accumulation (*30*). This signifies that accumulation may be a crucial computation for generating appropriate behavior based on noisy sensory information. The animal's needs also consist of real-time, fluctuating noisy information based on the animal's state or predictions. Utilizing accumulation to make decisions allows the animal to take appropriate actions based on this information, facilitating suitable behavioral choices. Also, recent research has shown that cognitive features such as needing and wanting could be separately dissociated in computational simulation (*8, 67*). To explore the question of whether accumulation is necessary under the constraints of our normative framework, we conducted a computer simulation to discern whether need requires accumulation for optimal eating behavior.

In an experiment based on need, two rules were put to the test. The first rule suggested that the predicted reward for the agent should change based on its behavioral states, while the second proposed that the predicted reward should not be affected by the state of behavior.

Following the first rule, the agent began its search for food from a starting point. As it got close to the food, the expected reward increased, causing the agent's need to decrease. Once the need fell below a threshold, the agent stopped and triggered a reset in reward expectation. This reset caused the need to rise rapidly again, albeit with a delay due to a behavioral change. As the agent resumed movement, the agent's internal variables reset again, causing the need to decrease even more rapidly and to a lower level than before. This resulted in a cycle of starts and stops, ultimately making the agent largely inefficient in the food-seeking behavior (Video S1, fig. S1B).

The results of the second rule were similar to those of the first, as the agent's initial behavior was the same. However, unlike the first rule, the system did not reset when interrupted by a decline in need. Consequently, the agent's deficit gradually increased, leading to a slow increase in need. Eventually, when the need surpassed the threshold, the agent started moving again. However, as soon as the agent moved, the need fell below the threshold, trapping it in a prolonged loop, similar to the first rule (Video S1, fig. S1C). While repeatedly performing go-and-stop actions may eventually lead to a reward, this alternating behavior can be inefficient and even dangerous. Energy may be wasted, and the agent may be exposed to predators, putting its survival at risk.

On the other hand, actions driven by motivation had a slower start by accumulating need. However, it exhibited strong persistence once motivation reached a threshold. Even though the

need decreases (or even goes to zero) as the agent gets close to the food, the motivation remains above the threshold due to the accumulation of need. This unwavering motivation successfully guided the agent towards eating food, surpassing the performance of the need model (Video S1, fig. S1D and E). These simulation results underscore the advantage of motivation-driven behavior over solely need-based behavior.

Therefore, motivation should be optimally generated as the integration of need. Motivation is also influenced by an integrated affecting factor (a). For example, motivation does not accumulate before accessibility is granted (*24*) $(a = 0)$. Additionally, motivation changes based on factors, such as satiety, valence, and memory (*24, 64*) $(a \geq 0)$. Therefore, the food-directed motivation at a specific time point $(M_f(t))$ can be expressed as the integral of the product between the integrated affecting factor and need. Finally, a leaky integrator was included to account for factors (mentioned above) that continuously devalue motivation in an animal. Note that in goal-directed motivation, the motivational state becomes 0 in situations where the goal becomes unattainable (either due to an abandoned moment, inaccessible moment, or depletion of food where there is no more food to consume). Actual neurons lose a certain amount of energy while storing information due to voltage or current leakage (*65*). Leaky integrate and fire (LIF) neuron models apply these leaks to explain the activity of real neurons and are frequently used in biophysics (*42, 43*). Without such leakage, if the accumulation of need continued persistently, motivation would always remain at a high value. To address this inconsistency, we aim to introduce the leaky integrate model here, seeking to explain the process by which motivation is generated through the accumulation of need.

$$M_f(t) = \int_{t_0}^{t} [a \times N_f(t) - Leak] \quad \text{(Equation S14)}$$

When sufficient motivation accumulates and surpasses a certain threshold ($K \geq 0$), behavior activity starts manifesting (*28*). Therefore, behavior outcome at a certain time point (t), can be written as follows:

$$B_f(t) = M_f(t) - K \quad \text{(Equation S15)}$$

Different Behavior Phenotypes of Stimulating AgRP Neurons in Different Experimental Settings

We speculate that the different behavior phenotypes of activating AgRP neurons in previous studies and our studies might be due to the different experimental settings the mice were in. One of the earliest experiments was from Dr. Sternson's group. They showed that activating AgRP neurons leads to an initiation of eating behavior and a latency to start eating can be observed.

In Dr. Sternson's group (*55*) the methods for assessing latency to feed are as follows.

Components for food consumption and photostimulation.

In the home cage, mice had ad-libitum access to mouse chow (PicoLab Rodent Diet 20, 5053 tablet, TestDiet). For behavioral testing, mice were transferred into feeding cages (Coulbourn Instruments) and supplied with food pellets (20 mg each) of identical composition to the food in the home cage, delivered by an automatic pellet dispenser. Pellet removal was sensed by the offset of a beam break and an additional pellet was administered after a delay (10 s). Food consumption was continuously monitored by computer using Graphic State (Coulbourn Instruments).

In our study, in supplementary video #5, mice were placed in a cage that positioned them directly in front of a cheese-flavored snack, which is very palatable to the mice. This snack fills up half of the cage and has a strong scent that mice can evidently recognize.

Therefore, when the need for food was increased by activating AgRP neurons, our mice could consume the highly palatable food immediately, which was clearly recognized as food was right in front of them. Conversely, in Dr. Sternson's paper, the mice would need to accumulate a higher amount of 'need' which would motivate them to search for the tiny unpalatable 20mg food pellet in a larger space (presumably a typical operant chamber which is 6~10 times the size of our optogenetic experiment). After initiating the search for food, a substantial amount of time will be required to discover the small pellet. We think that these differences in the experimental settings made a difference in the integrating affecting factors (a) in our motivation equation or the behavior threshold (K) in our behavior equation.

$$M_f(t)=\int_{t_0}^{t}[a \times N_f(t) - Leak]$$

$$B_f(t) = M_f(t) - K$$

This difference between the two experiments would have led to the difference in inclination of the increase of motivation. The experimental setting in Dr. Sternson's paper might have had a small effect on a and made a high K while our experimental setting might have had a big effect on a and made a low K.

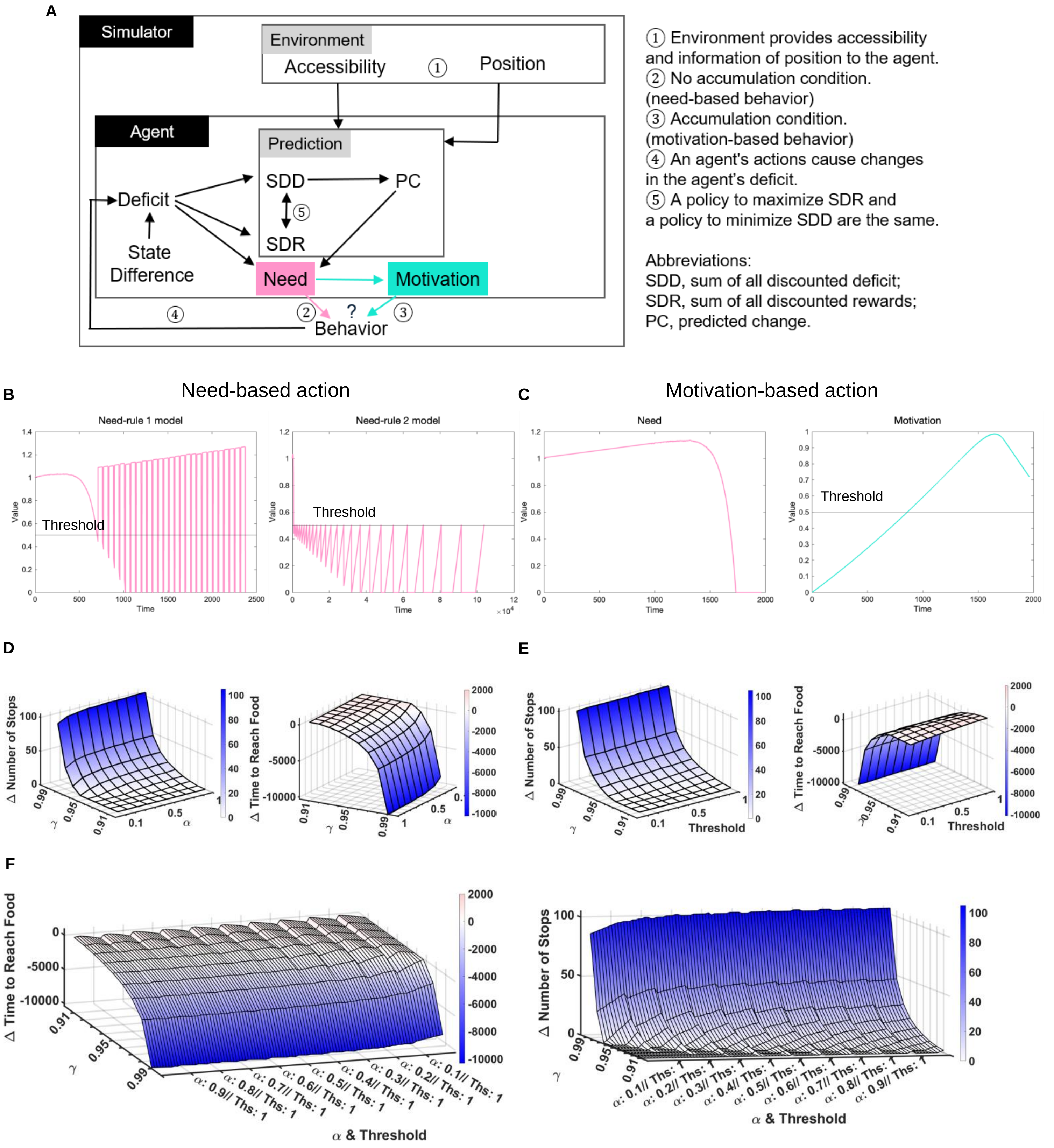

Fig. S1 | **Simulations Demonstrate that Accumulation of Need is Required for Survival.**
A, Schematic of simulation. **B,** Time courses of Need-rule 1 (left) and Need-rule 2 (right) model for the entire progression in the model where behavior is generated by need. Both models exhibit an inefficient pattern of repeated go and stop based on the threshold. The black line is the threshold where behavior is generated. **C,** Time courses of Need (left) and Motivation (right) for the entire progression in the model where behavior is generated by motivation. The black line is the threshold where behavior is generated. **D, E, F,** Simulation results of the difference of number of stops or time to reach food (△ Number of stops = stops in need-based model – stops in motivation-based model, △ Time to Reach Food = Time in need-based model – time in motivation based model). **D,** Results of changing discounting factor (γ) and integrated affecting factors (α). **E,** Results of changing γ and threshold. **F,** Comprehensive results of γ, α and threshold. α and threshold values are dissected in 0.1 between ticks (e.g. α: 0.8 // Ths: 1 to α: 0.9 // Ths: 1 is α: 0.8 // Ths: 0.1, α: 0.8 // Ths: 0.2, … α: 0.8 // Ths: 0.9, α: 0.8 // Ths: 1).

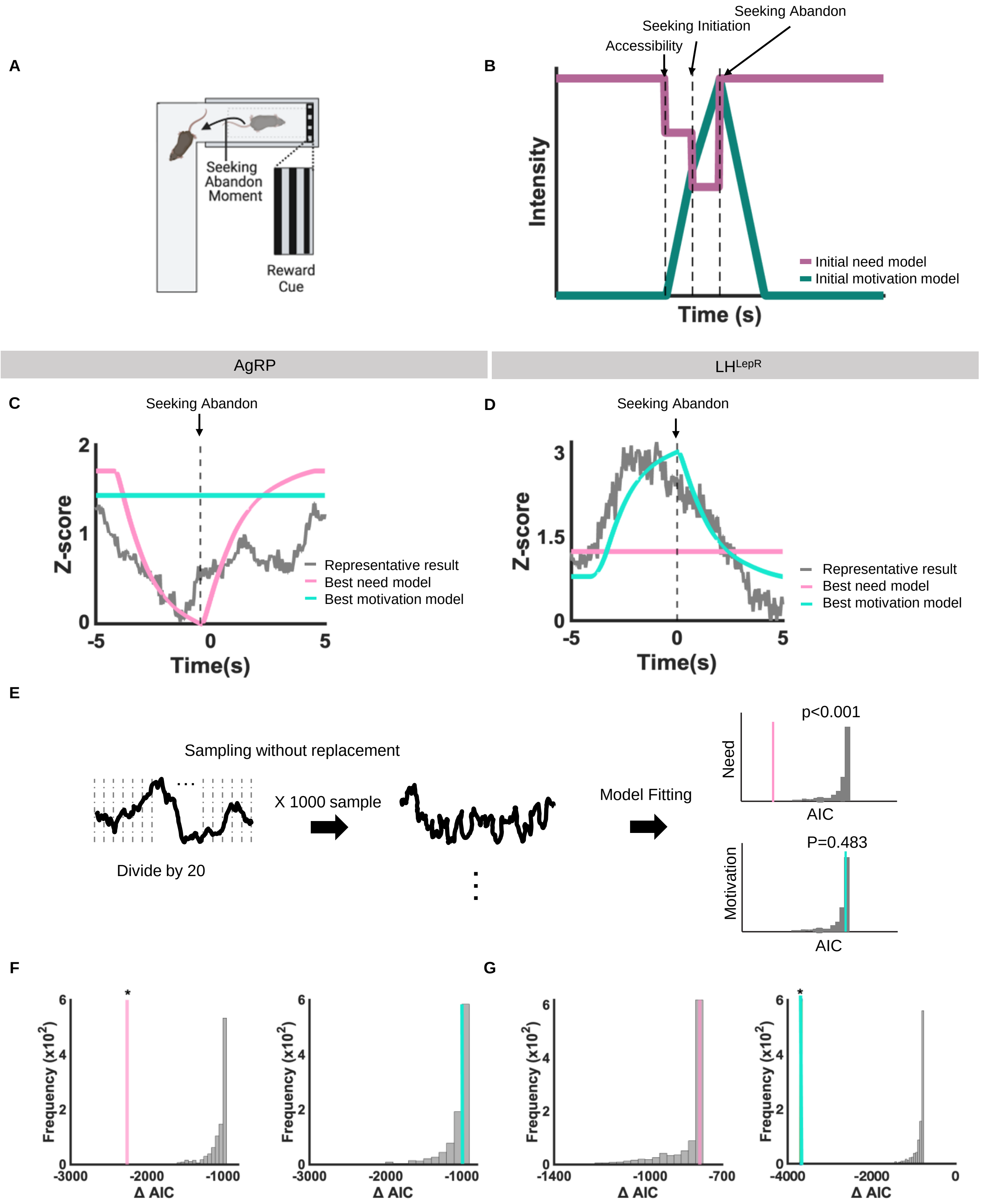

Fig. S2 | **Individual Trial Model Fitting Results Dissociates Need and Motivation in the Hypothalamic Neurons. A,** Schematic of behavioral paradigm and event moment. **B,** Schematic of initial model of neural activity from need (pale-violet-red) and motivation (teal) neurons. **C, D,** Individual trial fitting of neural activity. Best fit need neural activity model is pink and best fit motivation neural activity model is turquoise. Gray is neural activity (normalized Z-score). Dotted line indicate seeking abandon moment. **C,** Neural activity of AgRP neurons, **D,** Neural activity of LH^{LepR} neurons. **E,** Schematic of individual trial analysis. **F, G,** Permutation test result of model fitting. Histogram is AIC from difference between model and sampled data set or experimental data set. **F,** Result from AgRP neural activity (N = 1, Trials 5, permuted 1000 times), **G,** LH^{LepR} neural activity (N = 1, Trials 5, permuted 1000 times). See Table S1 for statistics. The schematics in A were created using BioRender.

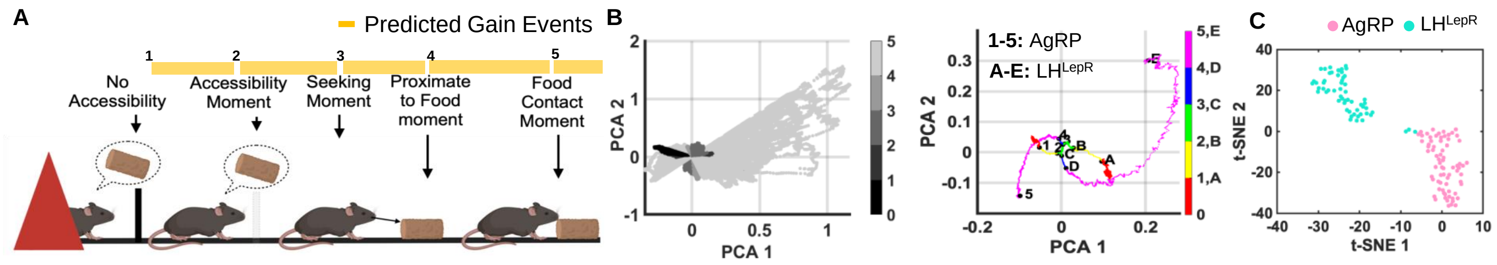

Fig. S3 | **Unbiased Dimensionality Reduction of Hypothalamic Neural Dynamics. A,** Schematic of behavioral paradigm of events that induce multi-predicted gain. **B,** PCA analysis of all neural trajectories (left) and average neural trajectories of AgRP neurons (1-5) and LH^{LepR} neurons (A-E), 0, Start of trial, 1, A, Accessibility, 2, B, Seeking initiation, 3, C, Proximate to food, 4, D, Contact, 5, E, End of trial (right). **C,** t-SNE analysis of all neural trajectories.

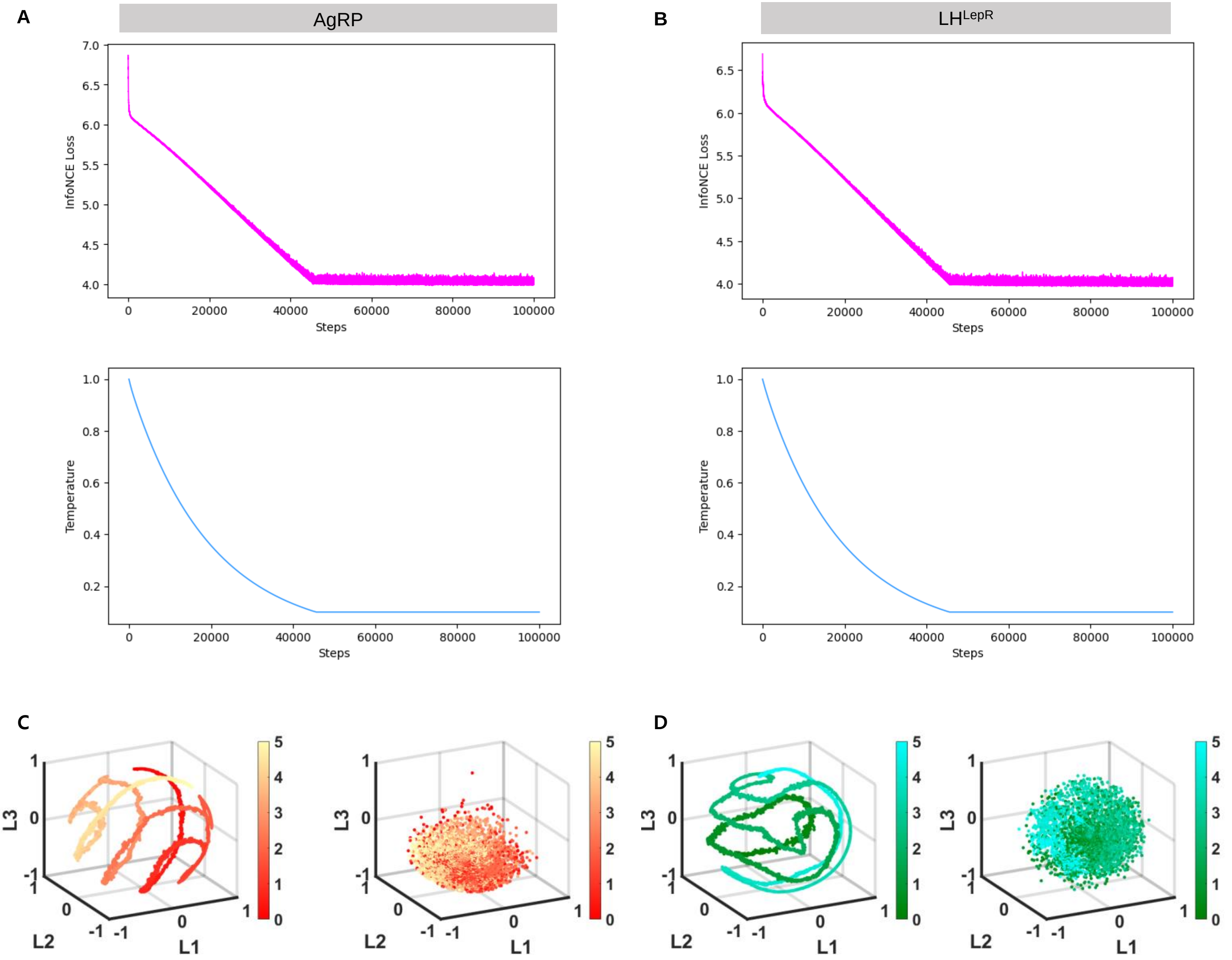

Fig. S4 | **CEBRA Analysis Show Different Hidden Embeddings in Hypothalamic Neurons.**

A, B, Convergence of training loss and temperature for model evaluation. **A,** AgRP neurons (N = 6, Trials = 72), **B,** LH^{LepR} neurons (N = 4, Trials = 56). **C, D,** Embeddings from CEBRA analysis from hypothalamic neurons. Left are embeddings from original labels, right are embeddings from shuffled labels. Colorbar is shown from 0 to 5 in order of predicted gain events (See the Methods). **C,** Embeddings from AgRP neurons (N = 6, Trials = 72). **D,** Embeddings from LH^{LepR} neurons (N = 4, Trials = 56). See Table S1 for statistics.

A

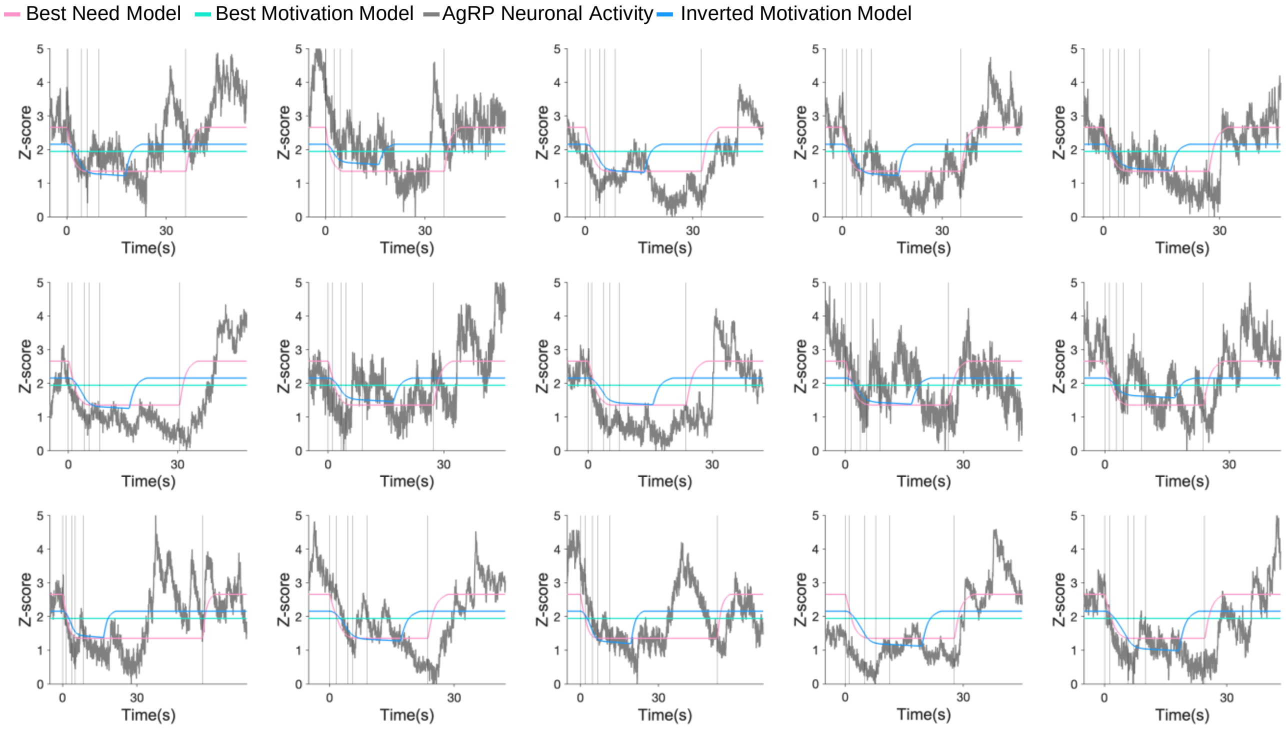

B

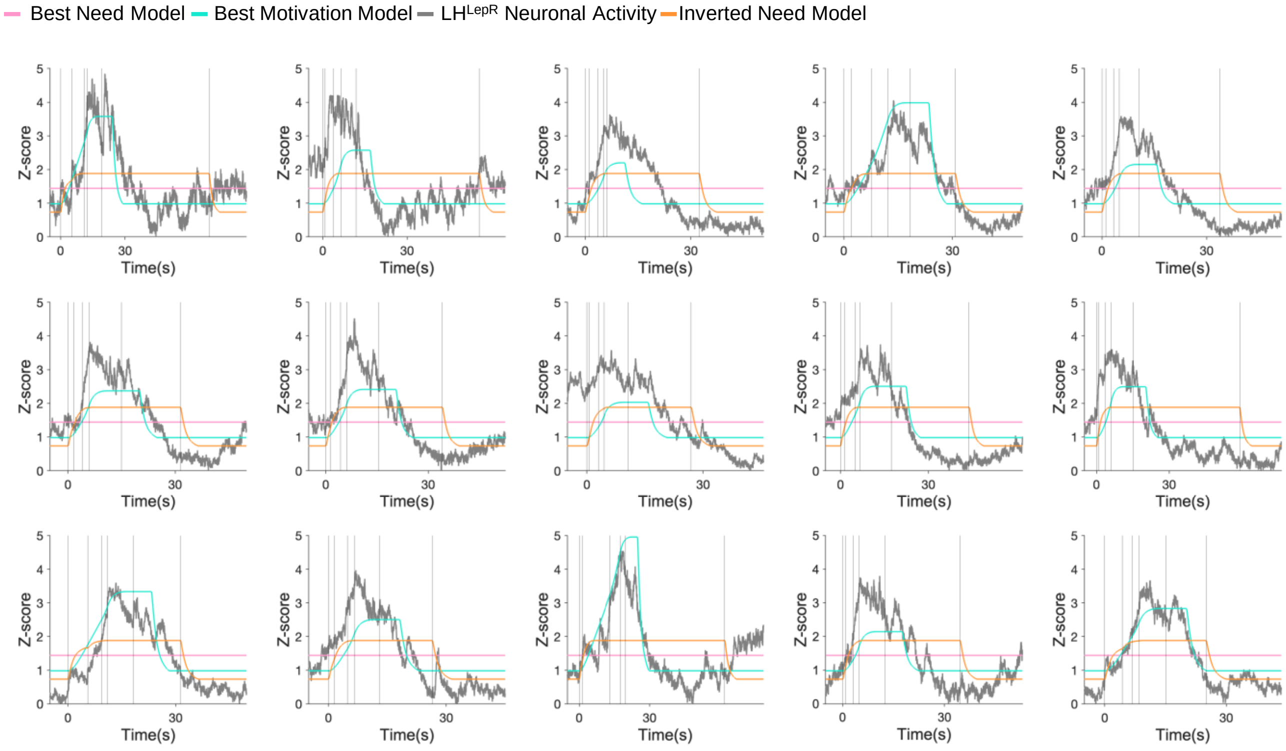

Fig. S5 | **Representative data from Cross-validated Models. A, B,** Representative cross-validated model fitting results between single trial neural activity data (normalized Z-score, gray) and best fit need neural activity model (pink), best fit motivation neural activity model (turquoise), best fit inverted motivation model (blue) or best fit inverted need model (orange) from an individual trial.Vertical lines along x-axis correspond to accessibility, seeking initiation, proximate to food, contact, inaccessibility moment in order. **A,** AgRP neurons, **B,** LH^{LepR} neurons.

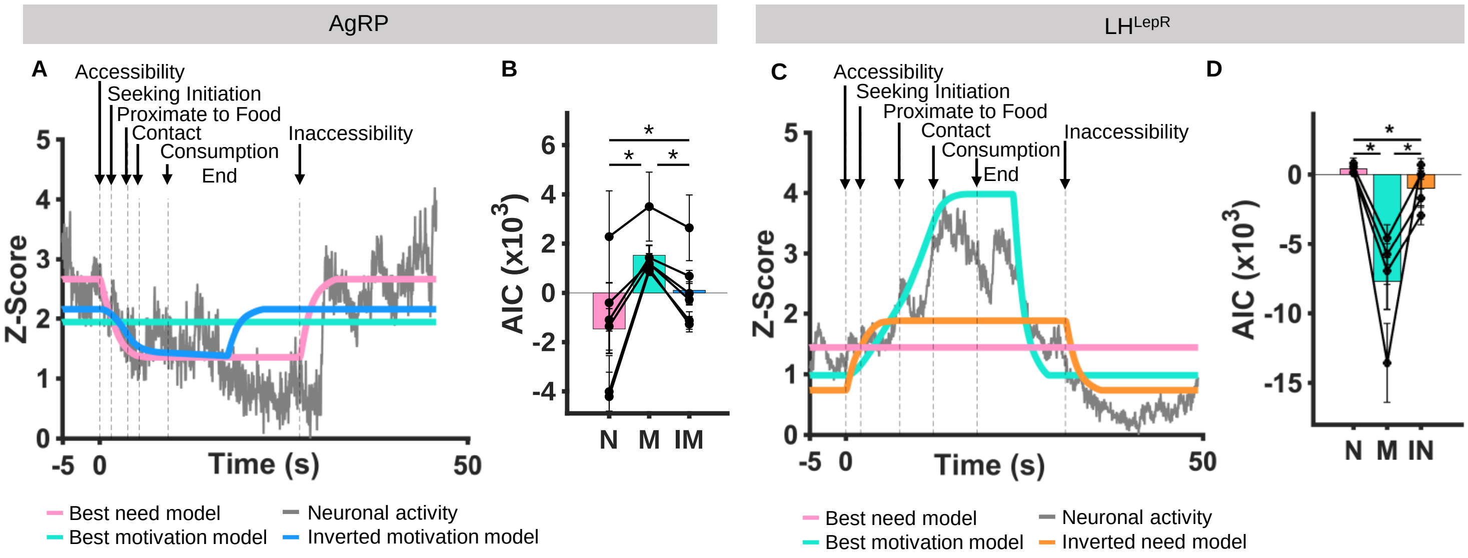

Fig. S6 | **Multi-Predicted Gain/Loss and Theory-Driven Models Dissociate AgRP and LH^{LepR} Neurons as Need and Motivation, Respectively, With Accounting Time Delay for Consumption End Behavior. A, C,** Representative cross-validated model fitting result between single trial neural activity data (normalized Z-score, gray) and best fit need neural activity model (pink), best fit motivation neural activity model (turquoise), best fit inverted motivation model (blue) or best fit inverted need model (orange) from an individual trial. **A,** Neural activity from AgRP neurons (N= 6, Trials= 56). **C,** Neural activity from LH^{LepR} neurons (N = 4, Trials = 50). **B, D,** Quantification of AIC between neural activity and best fit need neural activity model (pink), best fit motivation neural activity model (turquoise), best fit inverted motivation model (blue) or best fit inverted need model (orange). **B,** Quantification of AIC from AgRP neuronal behavior activation (N=6). **D,** Quantification of AIC from LH^{LepR} neuronal behavior activation (N=4). Error bar in each point represent s.e.m. for test set data in each individual mice.

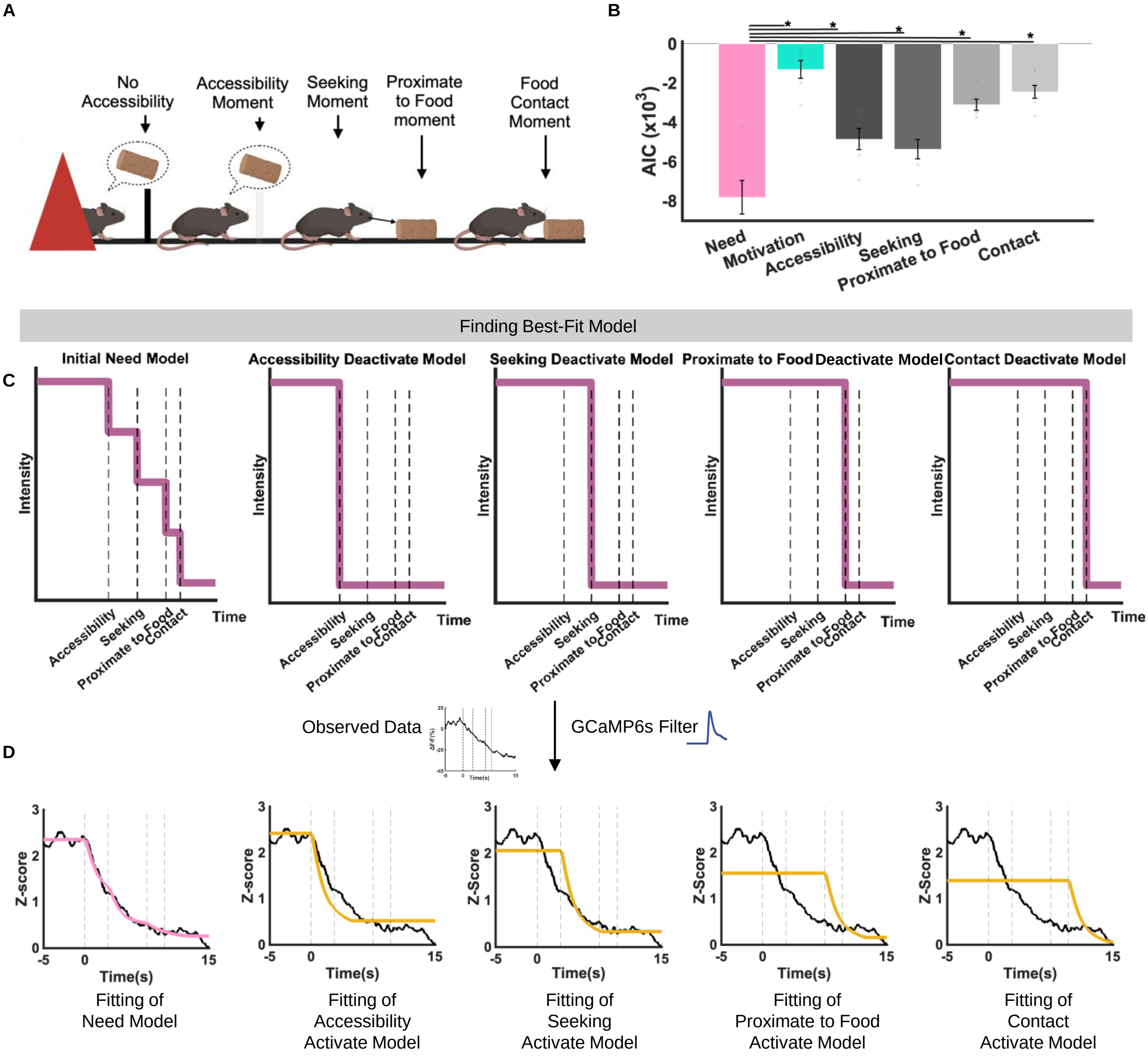

Fig. S7 | **Model Fitting Between Candidate Models and AgRP Neural Activity Show Need Neural Activity Model as Best-Fit Model. A,** Schematic of behavioral paradigm and event moment during multi-predicted gain test. **B,** Quantification of AIC between AgRP neural activity and candidate models (N = 6). **C,** Schematic of model prediction from each candidate model. **D,** Representative model fitting between average trace of AgRP neural activity (normalized Z-score) and candidate models from 1 mouse. Dotted line along x-axis correspond to accessibility, seeking, proximate to food, contact moment in order. Data are mean ± s.e.m. See Table S1 for statistics. The schematics in A were created using BioRender.

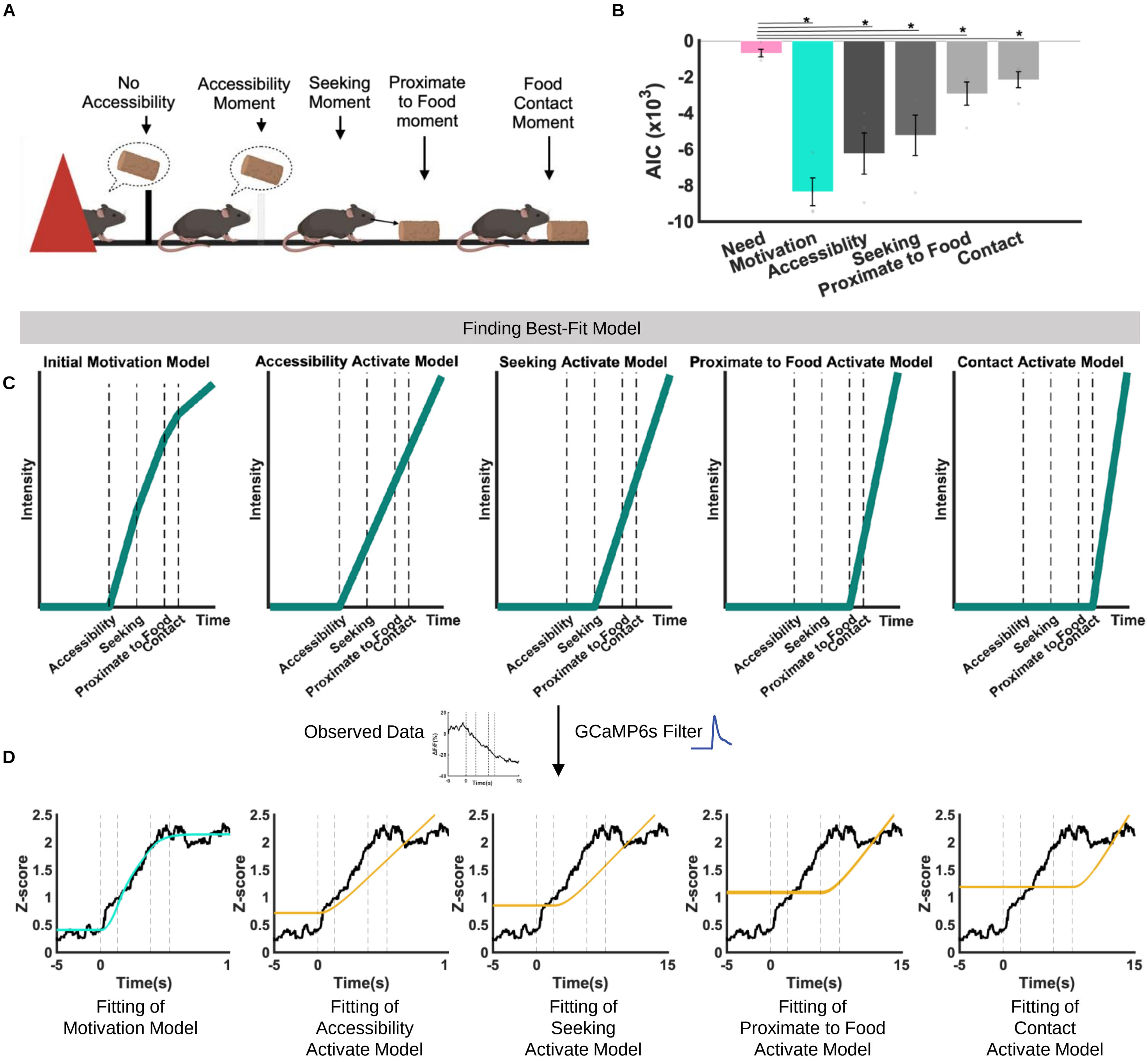

Fig. S8 | **Model Fitting between Candidate Models and LH^{LepR} Neural Activity Show Motivation Neural Activity Model as Best-Fit. A,** Schematic of behavioral paradigm and event moment during multi-predicted gain test. **B,** Quantification of AIC between LH^{LepR} neural activity and candidate models (N = 4). **C,** Schematic of model prediction from each candidate model. **D,** Representative model fitting between average trace of LH^{LepR} neural activity (normalized Z-score) and candidate models from 1 mouse. Dotted line along x-axis correspond to accessibility, seeking, proximate to food, contact moment in order. Data are mean ± s.e.m. See Table S1 for statistics. The schematics in A were created using BioRender.

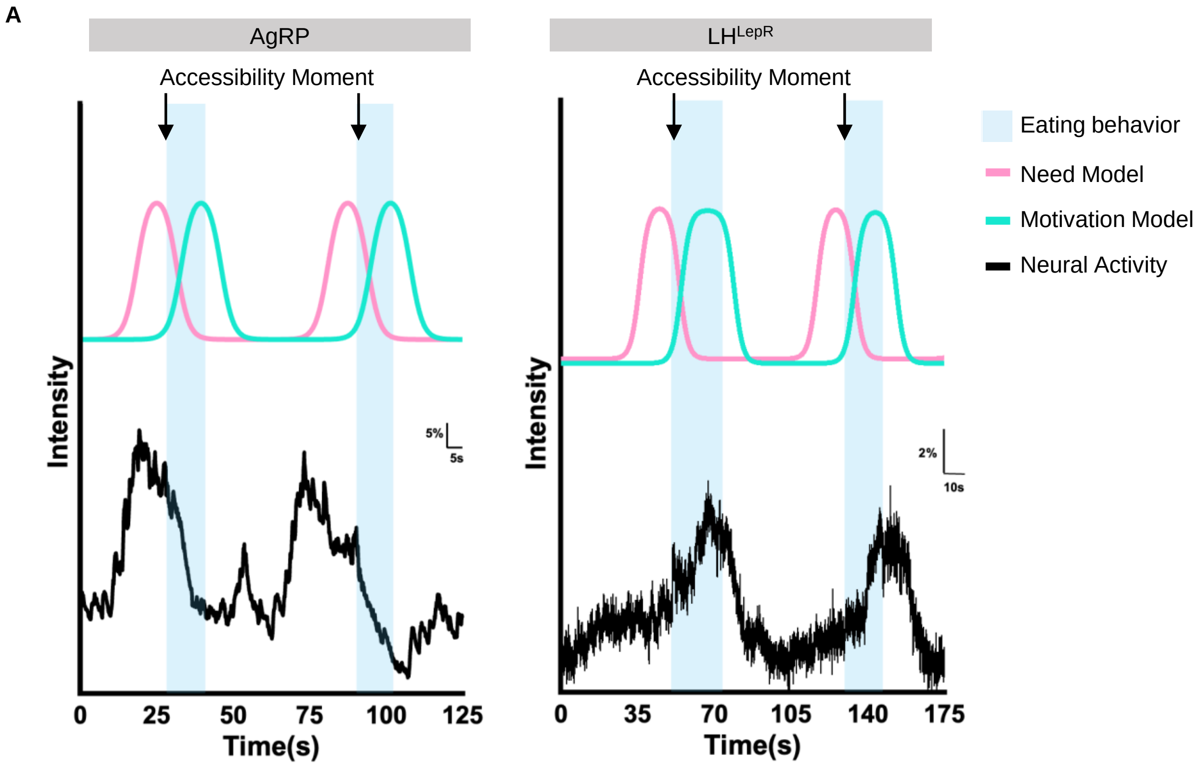

Fig. S9 | **Summary of the Temporal Dynamics of AgRP and LH^{LepR} Neurons Encoding Need and Motivation, Respectively. A,** Schematic of AgRP neurons (black, left) and LH^{LepR} neurons (black, right) encoding need (pink) and motivation (turquoise), respectively.

Table S1. Parameter values of models

Figure	Protocol	Best-fit parameters (Mean)	Best-fit parameters (Standard deviation)	Data type
Fig. 2G-I	AgRP-Predicted Gain Test 1	0.0303, 0.0259	0.0109, 0.0177	Average responses for each condition
Fig. 2J-L	LepR-Predicted Gain Test 1	0.0097, 0.0011	0.0024 1.1330e-04	Average responses for each condition
Fig. 2O-Q	AgRP-Predicted Gain Test 2	0.0328,0.0288	0.0110, 0.0162	Average responses for each condition
Fig. 2R-T`	LepR-Predicted Gain Test 2	0.0059, 0.0019	5.6829e-04 1.3376e-05	Average responses for each condition
Fig. 3C-E	AgRP-Predicted Loss Test 1	0.0094, 0.0135	0.0022, 0.0065	Average responses for each condition
Fig. 3F-H	LepR-Predicted Loss Test 1	Omitted due to no best-fit model		
Fig. 3K-M	AgRP-Predicted Loss Test 2	0.0304, 0.0184, 0.0174	0.0035, 0.0067, 0.0193	Average responses for each condition
Fig. 3N-P	LepR-Predicted Loss Test 2	0.0185, 0.0004, 0.0058	0.0075 1.6284e-04 8.4394e-04	Average responses for each condition
Fig. 4C	AgRP-Multi Predicted Gain/Loss Test	0.0469, 0.0091, 0.0092, 0.0068, 0.4998	0.0074,0.0070,0.0050,0.0035,2.7101e-04	Individual trial
Fig. 4F	LepR-Multi Predicted Gain/Loss Test	0.0297,6.793.5e-05,7.1569e-05,2.2410e-04,5.6654e-05	0.0097,6.4322e-05,7.4817e-05,2.0818e-04,8.8107e-05	Individual trial
Fig. S6	AgRP-Multi Predicted Gain/Loss Test (End time delay)	0.0469,0.0091,0.0092,0.0068,0.4999	0.0074,0.0070,0.0050,0.0035,1.5537e-04	Individual trial
Fig. S6	LepR-Multi Predicted Gain/Loss Test (End time delay)	0.0271,7.7263e-05,6.8153e-05,2.2180e-04,2.2509e-05,137.6652	0.0096,1.2374e-07,5.0652e-05,6.4834e-05,2.9922e-05,54.6726	Individual trial
Fig. S7	AgRP-Individual Event Based Candidate Models	0.0404, 0.0182, 0.0084, 0.0041, 0.0026	0.0069, 0.0088, 0.0068, 0.0052, 0.0025	Average responses for each condition
Fig. S8	LepR-Individual Event Based Candidate Models	0.0061 3.5540e-04 1.0676e-04 1.5452e-04 1.2222e-04	0.0027 2.5208e-04 4.9909e-05 1.6183e-04 1.2255e-04	Average responses for each condition

Values are in the order of parameters for the models. Data to replicate p-values are provided in Dryad (See Acknowledgements).

Tables S2. Summary of statistical analysis

Figure	Figure	Mouse	Virus	Mouse	Behavior test, Number of times performed (*N*)	Specific Figure	Statistics	N	Trials	p-value
Fig. 2	Fig. 2G-I	AgRP	GCaMP6s	AgRP4-3, AgRP4-5, AgRP5-3, AgRP7-2	Predicted Gain Test 1, *N*=1	Figure 2I	Friedman's Test	4	15/7/12/12	0.0455
	Fig.2J-L	LepR		LepR 20-4 LepR 26 LepR 40-1 LepR 43-2		Figure 2L	Friedman's Test	4	13/29/12/13	0.0455
	Fig. 2O-Q	AgRP		AgRP4-3, AgRP4-5, AgRP5-3, AgRP7-2	Predicted Gain Test 2, *N*=1	Figure 2Q	Friedman's Test	4	12/12/12/12	0.0455
	Fig. 2R-T	LepR		LepR 20-4 LepR 26 LepR 38 LepR 40-1		Figure 2T	Friedman's Test	4	12/12/12/12	0.0455
Fig. 3	Fig. 3C-E	AgRP	GCaMP6s	AgRP4-3, AgRP4-5, AgRP5-3, AgRP7-2 AgRP8-1	Predicted Loss Test 1, *N*=1	Figure 3E	Friedman's Test	5	15/15/15/15/15	0.0455
	Fig. 3F-H	LepR		LepR 20-4 LepR 26 LepR 40-1 LepR 43-2		Figure 3H	Friedman's Test	4	15/15/15/15	1
	Fig. 3K-M	AgRP		AgRP4-3, AgRP4-5, AgRP5-3, AgRP7-2	Predicted Loss Test 2, *N*=1	Figure 3M	Friedman's Test	4	2/7/5/6	0.0455
	Fig. 3N-P	LepR		LepR 20-4 LepR 26 LepR 27 LepR 40-1		Figure 3P	Friedman's Test	5	1/4/1/7/4	0.0253

				LepR 43-2						
Fig. 4	Fig. 4D	AgRP	GCaMP6s	AgRP4-3,<br>AgRP4-5,<br>AgRP5-3,<br>AgRP7-2<br>AgRP8-1<br>AgRP 8-3	Multi-Predicted Gain/Loss Test, *N*=1	Figure 4D	Friedman's Test	6	15/12/11/7/12/9	Need-Motivation: 0.0143<br>Need-Inverted Motivation: 0.0143<br>Motivation-Inverted-Motivation:0.0143
	Fig. 4F	LepR		LepR 20-4<br>LepR 26<br>LepR 40-1<br>LepR 43-2		Figure 4F	Friedman's Test	4	14/12/9/15	Need-Motivation: 0.0455<br>Need-Inverted Need: 0.0455<br>Motivation-Inverted Need: 0.3173
	Fig. 4H	AgRP		AgRP4-3,<br>AgRP4-5,<br>AgRP5-3,<br>AgRP7-2<br>AgRP8-1<br>AgRP 8-3		Figure 4H				Simple Test: $2.7e^{-5}$<br>Complex Test: 0.0005
	Fig. 4I	LepR		LepR 20-4<br>LepR 26<br>LepR 40-1<br>LepR 43-2		Figure 4I				Simple Test: 0.39<br>Complex Test: 0.034
Fig.5	Fig. 5E-F	AgRP	ChR2	AgRP 2-1<br>AgRP 2-2<br>AgRP 7-1<br>AgRP 7-2<br>AgRP 10-1<br>AgRP 10-2	Eating Evoke Test, *N*=1	Figure 5F	Paired T-Test	6	6/6/6/6/6/6	Pre-Laser: 0.000169<br>Pre-Post:<br>0.000000136
	Fig. 5G-H	LepR		LepR 43-4<br>LepR 51-1<br>LepR 51-5<br>LepR 51-6<br>LepR 53-3<br>LepR 53-4		Figure 5H	Paired T-Test	6	10/9/10/11/11/14	Pre-Laser: 0.0014<br>Laser-Post: 0.0013

	Fig. 5J	AgRP		AgRP 2-1<br>AgRP 2-2<br>AgRP 7-1<br>AgRP 7-2<br>AgRP 10-1<br>AgRP 10-2		Figure 5J	Friedman's Test	6	6/6/6/6/6/6	0.0143
	Fig. 5L	LepR		LepR 43-4<br>LepR 51-1<br>LepR 51-5<br>LepR 51-6<br>LepR 53-3<br>LepR 53-4		Figure 5L	Friedman's Test	6	10/9/10/11/11/14	0.0143
Fig. S2	Fig. S2F	AgRP	GCaMP6s	AgRP 4-5	Predicted Loss Test 3, N=1	Fig. S2F	Permutation Test	1	5	Red Line <0.001<br>Green Line = 0.483
	Fig. S2G	LepR	GCaMP6s	LepR 38		Fig. S2G	Permutation Test	1	5	Red Line = 0.503<br>Green Line = <0.001
Fig. S6	Fig. S6B	AgRP,	GCaMP6s	AgRP4-3,<br>AgRP4-5,<br>AgRP5-3,<br>AgRP7-2<br>AgRP8-1<br>AgRP 8-3,	Multi-Predicted Gain/Loss test, N=1	Fig. S6B	Friedman's Test	6	15/12/11/7/12/9	Need-Motivation: 0.0143<br>Need-Inverted Motivation: 0.014<br>Motivation-Inverted Motivation: 0.0143
Fig. S6	Fig. S6D	LepR,	GCaMP6s	LepR 20-4<br>LepR 26<br>LepR 40-1<br>LepR 43-2	Multi-Predicted Gain/Loss test, N=1	Fig. S6D	Friedman's Test	4	14/12/9/15	Motivation-Need: 0.0455<br>Motivation-Inverted Need: 0.0455<br>Need Inverted-Need:0.0455
Fig. S7	Fig. S7B	AgRP	GCaMP6s	AgRP4-3,<br>AgRP4-5,<br>AgRP5-3,<br>AgRP7-2<br>AgRP8-1<br>AgRP 8-3	Multi-Predicted Gain test, N=1	Fig. S6B	Friedman's Test	6	15/12/11/7/12/9	Comparison with Need -<br>Motivation: 0.0143<br>Accessibility: 0.0143<br>Seeking: 0.0143<br>Proximate to Food: 0.0143

										Contact: 0.0143
Fig. S8	Fig.S8B	LepR	GCaMP6s	LepR 20-4<br><br>LepR 26<br><br>LepR 40-1<br><br>LepR 43-2	Multi-Predicted Gain test, N=1	Fig. S7B	Friedman's Test	4	14/12/9/15	Comparison with Motivation -<br><br>Need: 0.0455<br><br>Accessibility: 0.0455<br><br>Seeking: 0.0455<br><br>Proximate to Food: 0.0455<br><br>Contact: 0.0455

Movies S1 to S5

Movie S1. Selection of Need-Driven Behavior Policy from Motivation-Driven Behavior Policy Using Computer Simulation.

Movie S2. CEBRA Analysis Reveals Different Latent Features in AgRP and LH^{LepR} Neurons.

Movie S3. Neural Activity Model Prediction of AgRP and LH^{LepR} Neurons Reveals Role as Need and Motivation, Respectively.

Movie S4. Behavior Activity Prediction of AgRP and LH^{LepR} Neurons Reveals Role as Need and Motivation, Respectively.

Movie S5. Summary of Temporal Dissociation of the Role of Hypothalamic Neurons as Need and Motivation.

REFERENCES AND NOTES

1. W. B. Cannon, *The Wisdom of the Body* (W W Norton & Co, 1932), pp. 312–312.

2. W. E. Allen, L. A. DeNardo, M. Z. Chen, C. D. Liu, K. M. Loh, L. E. Fenno, C. Ramakrishnan, K. Deisseroth, L. Luo, Thirst-associated preoptic neurons encode an aversive motivational drive. *Science* **357**, 1149–1155 (2017).

3. Y. H. Lee, M. Kim, M. Lee, D. Shin, D. S. Ha, J. S. Park, Y. B. Kim, H. J. Choi, Food craving, seeking, and consumption behaviors: Conceptual phases and assessment methods used in animal and human studies. *J. Obes. Metab. Syndr.* **28**, 148–157 (2019).

4. G. Pezzulo, F. Rigoli, K. Friston, Active Inference, homeostatic regulation and adaptive behavioural control. *Prog. Neurobiol.* **134**, 17–35 (2015).

5. K. Juechems, J. Balaguer, S. Herce Castañón, M. Ruz, J. X. O'Reilly, C. Summerfield, A network for computing value equilibrium in the human medial prefrontal cortex. *Neuron* **101**, 977–987.e3 (2019).

6. C. L. Hull, *Principles of Behavior: An Introduction to Behavior Theory* (Appleton-Century, 1943), p. 422.

7. K. C. Berridge, Motivation concepts in behavioral neuroscience. *Physiol. Behav.* **81**, 179–209 (2004).

8. J. Bosulu, G. Pezzulo, S. Hétu, A computational account of needing and wanting. bioRxiv, 2022.2010.2024.513547 [Preprint] (2023). https://doi.org/10.1101/2022.10.24.513547.

9. O. J. Hulme, T. Morville, B. Gutkin, Neurocomputational theories of homeostatic control. *Phys. Life Rev.* **31**, 214–232 (2019).

10. H. R. Kim, A. N. Malik, J. G. Mikhael, P. Bech, I. Tsutsui-Kimura, F. Sun, Y. Zhang, Y. Li, M. Watabe-Uchida, S. J. Gershman, N. Uchida, A unified framework for dopamine signals across timescales. *Cell* **183**, 1600–1616.e25 (2020).

11. J. Cox, A. R. Minerva, W. T. Fleming, C. A. Zimmerman, C. Hayes, S. Zorowitz, A. Bandi, S. Ornelas, B. McMannon, N. F. Parker, I. B. Witten, A neural substrate of sex-dependent modulation of motivation. *Nat. Neurosci.* **26**, 274–284 (2023).

12. K. Choi, E. Piasini, E. Díaz-Hernández, L. V. Cifuentes, N. T. Henderson, E. N. Holly, M. Subramaniyan, C. R. Gerfen, M. V. Fuccillo, Distributed processing for value-based choice by prelimbic circuits targeting anterior-posterior dorsal striatal subregions in male mice. *Nat. Commun.* **14**, 1920 (2023).

13. M. Keramati, B. Gutkin, Homeostatic reinforcement learning for integrating reward collection and physiological stability. *eLife* **3**, (2014).

14. C. Gizowski, C. Zaelzer, C. W. Bourque, Clock-driven vasopressin neurotransmission mediates anticipatory thirst prior to sleep. *Nature* **537**, 685–688 (2016).

15. C. Deans, Biological prescience: The role of anticipation in organismal processes. *Front. Physiol.* **12**, 672457 (2021).

16. V. Augustine, S. K. Gokce, Y. Oka, Peripheral and central nutrient sensing underlying appetite regulation. *Trends Neurosci.* **41**, 526–539 (2018).

17. Y. Livneh, A. U. Sugden, J. C. Madara, R. A. Essner, V. I. Flores, L. A. Sugden, J. M. Resch, B. B. Lowell, M. L. Andermann, Estimation of current and future physiological states in insular cortex. *Neuron* **105**, 1094–1111.e10 (2020).

18. F. Reed, S. H. Lockie, A. Reichenbach, C. J. Foldi, Z. B. Andrews, Appetite to learn: An allostatic role for AgRP neurons in the maintenance of energy balance. *Curr. Opin. Endocr. Metab. Res.* **24**, 100337 (2022).

19. M. Torigoe, T. Islam, H. Kakinuma, C. C. A. Fung, T. Isomura, H. Shimazaki, T. Aoki, T. Fukai, H. Okamoto, Zebrafish capable of generating future state prediction error show improved active avoidance behavior in virtual reality. *Nat. Commun.* **12**, 5712 (2021).

20. C. A. Zimmerman, Y. C. Lin, D. E. Leib, L. Guo, E. L. Huey, G. E. Daly, Y. Chen, Z. A. Knight, Thirst neurons anticipate the homeostatic consequences of eating and drinking. *Nature* **537**, 680–684 (2016).

21. D. S. Ramsay, S. C. Woods, Clarifying the roles of homeostasis and allostasis in physiological regulation. *Psychol. Rev.* **121**, 225–247 (2014).

22. C. L. Chen, F. Aymanns, R. Minegishi, V. D. V. Matsuda, N. Talabot, S. Günel, B. J. Dickson, P. Ramdya, Ascending neurons convey behavioral state to integrative sensory and action selection brain regions. *Nat. Neurosci.* **26**, 682–695 (2023).

23. T. Akam, I. Rodrigues-Vaz, I. Marcelo, X. Zhang, M. Pereira, R. F. Oliveira, P. Dayan, R. M. Costa, The anterior cingulate cortex predicts future states to mediate model-based action selection. *Neuron* **109**, 149–163.e7 (2021).

24. *The Interoceptive Mind: From Homeostasis to Awareness*, M. Tsakiris, H. De Preester Eds. (Oxford Univ. Press, 2018).

25. B. S. McEwen, J. C. Wingfield, The concept of allostasis in biology and biomedicine. *Horm. Behav.* **43**, 2–15 (2003).

26. R. A. Hinde, Ethological models and the concept of 'drive'. *Br. J. Philos. Sci.* **6**, 321–331 (1956).

27. D. S. Lehrman, A critique of Konrad Lorenz's theory of instinctive behavior. *Q. Rev. Biol.* **28**, 337–363 (1953).

28. R. Dawkins, A threshold model of choice behaviour. *Anim. Behav.* **17**, 120–133 (1969).

29. K. Z. Lorenz, The comparative method in studying innate behavior patterns, in *Physiological Mechanisms in Animal Behavior. (Society's Symposium IV.)*, (Academic Press, 1950), pp. 221–268.

30. J. D. Davidson, A. El Hady, Foraging as an evidence accumulation process. *PLoS Comput. Biol.* **15**, e1007060 (2019).

31. D. O. Hebb, *The Organization of Behavior: A Neuropsychological Theory,* (Wiley, 1949), p. 335.

32. S. Luquet, F. A. Perez, T. S. Hnasko, R. D. Palmiter, NPY/AgRP neurons are essential for feeding in adult mice but can be ablated in neonates. *Science* **310**, 683–685 (2005).

33. Q. Gao, T. L. Horvath, Neurobiology of feeding and energy expenditure. *Annu. Rev. Neurosci.* **30**, 367–398 (2007).

34. T. M. Hahn, J. F. Breininger, D. G. Baskin, M. W. Schwartz, Coexpression of Agrp and NPY in fasting-activated hypothalamic neurons. *Nat. Neurosci.* **1**, 271–272 (1998).

35. J. D. Deem, C. L. Faber, G. J. Morton, AgRP neurons: Regulators of feeding, energy expenditure, and behavior. *FEBS J.* **289**, 2362–2381 (2022).

36. I. C. Alcantara, A. P. M. Tapia, Y. Aponte, M. J. Krashes, Acts of appetite: Neural circuits governing the appetitive, consummatory, and terminating phases of feeding. *Nat. Metab.* **4**, 836–847 (2022).

37. Q. Liu, X. Yang, M. Luo, J. Su, J. Zhong, X. Li, R. H. M. Chan, L. Wang, An iterative neural processing sequence orchestrates feeding. *Neuron* **111**, 1651–1665.e5 (2023).

38. Y. H. Lee, Y. B. Kim, K. S. Kim, M. Jang, H. Y. Song, S. H. Jung, D. S. Ha, J. S. Park, J. Lee, K. M. Kim, D. H. Cheon, I. Baek, M. G. Shin, E. J. Lee, S. J. Kim, H. J. Choi, Lateral hypothalamic leptin receptor neurons drive hunger-gated food-seeking and consummatory behaviours in male mice. *Nat. Commun.* **14**, 1486 (2023).

39. S. Shin, I. J. You, M. Jeong, Y. Bae, X. Y. Wang, M. L. Cawley, A. Han, B. K. Lim, Early adversity promotes binge-like eating habits by remodeling a leptin-responsive lateral hypothalamus-brainstem pathway. *Nat. Neurosci.* **26**, 79–91 (2023).

40. M. E. Mazurek, J. D. Roitman, J. Ditterich, M. N. Shadlen, A role for neural integrators in perceptual decision making. *Cereb. Cortex* **13**, 1257–1269 (2003).

41. S. Bitzer, H. Park, F. Blankenburg, S. J. Kiebel, Perceptual decision making: Drift-diffusion model is equivalent to a Bayesian model. *Front. Hum. Neurosci.* **8**, 102 (2014).

42. O. B. Artun, H. Z. Shouval, L. N. Cooper, The effect of dynamic synapses on spatiotemporal receptive fields in visual cortex. *Proc. Natl. Acad. Sci. U.S.A.* **95**, 11999–12003 (1998).

43. D. Millman, S. Mihalas, A. Kirkwood, E. Niebur, Self-organized criticality occurs in non-conservative neuronal networks during Up states. *Nat. Phys.* **6**, 801–805 (2010).

44. Y. Chen, R. A. Essner, S. Kosar, O. H. Miller, Y. C. Lin, S. Mesgarzadeh, Z. A. Knight, Sustained NPY signaling enables AgRP neurons to drive feeding. *eLife* **8**, (2019).

45. S. Rutherford, S. M. Kia, T. Wolfers, C. Fraza, M. Zabihi, R. Dinga, P. Berthet, A. Worker, S. Verdi, H. G. Ruhe, C. F. Beckmann, A. F. Marquand, The normative modeling framework for computational psychiatry. *Nat. Protoc.* **17**, 1711–1734 (2022).

46. E. B. Richman, N. Ticea, W. E. Allen, K. Deisseroth, L. Luo, Neural landscape diffusion resolves conflicts between needs across time. *Nature* **623**, 571–579 (2023).

47. T. T. Hills, P. M. Todd, D. Lazer, A. D. Redish, I. D. Couzin, Exploration versus exploitation in space, mind, and society. *Trends Cogn. Sci.* **19**, 46–54 (2015).

48. A. Dickinson, Actions and habits: The development of behavioural autonomy. *Philos. Trans. R. Soc. Lond. B Biol. Sci.* **308**, 67–78 (1985).

49. H. H. Yin, B. J. Knowlton, B. W. Balleine, Lesions of dorsolateral striatum preserve outcome expectancy but disrupt habit formation in instrumental learning. *Eur. J. Neurosci.* **19**, 181–189 (2004).

50. N. D. Daw, J. P. O'Doherty, Chapter 21 - Multiple systems for value learning, in *Neuroeconomics (Second Edition),* P. W. Glimcher, E. Fehr, Eds., (Academic Press, 2014), pp. 393–410.

51. N. M. Seel, Model-based learning: A synthesis of theory and research. *Educ. Technol. Res. Dev.* **65**, 931–966 (2017).

52. Y. Chen, Y. C. Lin, T. W. Kuo, Z. A. Knight, Sensory detection of food rapidly modulates arcuate feeding circuits. *Cell* **160**, 829–841 (2015).

53. A. Petzold, H. E. van den Munkhof, R. Figge-Schlensok, T. Korotkova, Complementary lateral hypothalamic populations resist hunger pressure to balance nutritional and social needs. *Cell Metab.* **35**, 456–471.e6 (2023).

54. M. R. Zimmer, A. H. O. Fonseca, O. Iyilikci, R. D. Pra, M. O. Dietrich, Functional ontogeny of hypothalamic Agrp neurons in neonatal mouse behaviors. *Cell* **178**, 44–59.e7 (2019).

55. Y. Aponte, D. Atasoy, S. M. Sternson, AGRP neurons are sufficient to orchestrate feeding behavior rapidly and without training. *Nat. Neurosci.* **14**, 351–355 (2011).

56. J. N. Siemian, M. A. Arenivar, S. Sarsfield, C. B. Borja, C. N. Russell, Y. Aponte, Lateral hypothalamic LEPR neurons drive appetitive but not consummatory behaviors. *Cell Rep.* **36**, 109615 (2021).

57. I. Aklan, N. Sayar Atasoy, Y. Yavuz, T. Ates, I. Coban, F. Koksalar, G. Filiz, I. C. Topcu, M. Oncul, P. Dilsiz, U. Cebecioglu, M. I. Alp, B. Yilmaz, D. R. Davis, K. Hajdukiewicz, K. Saito, W. Konopka, H. Cui, D. Atasoy, NTS catecholamine neurons mediate hypoglycemic hunger via medial hypothalamic feeding pathways. *Cell Metab.* **31**, 313–326.e5 (2020).

58. T. W. Chen, T. J. Wardill, Y. Sun, S. R. Pulver, S. L. Renninger, A. Baohan, E. R. Schreiter, R. A. Kerr, M. B. Orger, V. Jayaraman, L. L. Looger, K. Svoboda, D. S. Kim, Ultrasensitive fluorescent proteins for imaging neuronal activity. *Nature* **499**, 295–300 (2013).

59. H. Akaike, A new look at the statistical model identification. *IEEE Trans. Automat. Contr.* **19**, 716–723 (1974).

60. J. J. Orban de Xivry, P. Lefèvre, A switching cost for motor planning. *J. Neurophysiol.* **116**, 2857–2868 (2016).

61. S. Schneider, J. H. Lee, M. W. Mathis, Learnable latent embeddings for joint behavioural and neural analysis. *Nature* **617**, 360–368 (2023).

62. M. Friedman, The use of ranks to avoid the assumption of normality implicit in the analysis of variance. *J. Am. Stat. Assoc.* **32**, 675–701 (1937).

63. F. M. Toates, Homeostasis and drinking. *Behav. Brain Sci.* **2**, 95–102 (1979).

64. A. K. Seth, Interoceptive inference, emotion, and the embodied self. *Trends Cogn. Sci.* **17**, 565–573 (2013).

65. M. A. Apps, M. Tsakiris, The free-energy self: A predictive coding account of self-recognition. *Neurosci. Biobehav. Rev.* **41**, 85–97 (2014).

66. M. Hovd, R. R. Bitmead, Feedforward for stabilization in the presence of constraints. *J. Process Control* **22**, 659–665 (2012).

67. K. C. Berridge, Separating desire from prediction of outcome value. *Trends Cogn. Sci.* **27**, 932–946 (2023).
